# High-density multidistance fNIRS enhances detection of brain activity during a word-color Stroop task

**DOI:** 10.1117/1.NPh.12.3.035010

**Published:** 2025-09-02

**Authors:** Jessica E. Anderson, Laura B. Carlton, Sreekanth Kura, Walker J. O’Brien, De’Ja Rogers, Parisa Rahimi, Parya Y. Farzam, Muhammad H. Zaman, David A. Boas, Meryem A. Yücel

**Affiliations:** a Boston University, Neurophotonics Center, Biomedical Engineering, Boston, Massachusetts, United States; b Boston University, Department of Electrical and Computer Engineering, Boston, Massachusetts, United States; c Boston University, Questrom School of Business, Boston, Massachusetts, United States; d Boston University, Department of Biomedical Engineering, Boston, Massachusetts, United States

**Keywords:** functional near-infrared spectroscopy, diffuse optical tomography, high-density functional near-infrared spectroscopy, word–color Stroop, pre-frontal cortex

## Abstract

**Significance:**

Functional near-infrared spectroscopy (fNIRS) enables neuroimaging in scenarios where other modalities are less suitable, such as during motion tasks or in low-resource environments. Sparse fNIRS arrays with 30 mm channel spacing are widely used but have limited spatial resolution. High-density (HD) arrays with overlapping, multidistance channels improve sensitivity and localization but increase costs and setup times. A statistical comparison of HD and sparse arrays is needed for evaluating the benefits and trade-offs of HD arrays.

**Aim:**

This study provides a statistical comparison of HD and sparse fNIRS performance to inform array selection in future research.

**Approach:**

We measured prefrontal cortex (PFC) activation during congruent and incongruent word–color Stroop (WCS) tasks using both sparse and HD arrays for 17 healthy adult participants, comparing dorsolateral PFC channel and image results at the group level.

**Results:**

Although both arrays detected activation in channel space during incongruent WCS, channel and image space results demonstrated superior localization and sensitivity with the HD array for all WCS.

**Conclusion:**

Sparse channel data may suitably detect activation from cognitively demanding tasks, such as incongruent WCS. However, the HD array outperformed sparse in detecting and localizing brain activity in image space, particularly during lower cognitive load tasks, making it more suitable for neuroimaging applications.

## 1 Introduction

The use of fNIRS has grown exponentially since its inception,^1^^,^^2^ expanding our understanding of brain activity in a variety of contexts, such as in psychiatry,^3^^–^^7^ naturalistic environments,^8^ developmental research,^9^^–^^11^ low-resource contexts and global health,^11^^–^^17^ and for hyperscanning studies.^18^^–^^21^ Benefits of functional near-infrared spectroscopy (fNIRS) over other neuroimaging modalities are well-defined for its comparative motion artifact resistance, temporal resolution, portability and wearability, operational requirements and ease of use, comfort, and general ecological validity.^22^

The traditional optode layout employed by most commercially available fNIRS systems and studies arranges sources and detectors in a nonoverlapping, 30 mm grid pattern. This low-density or sparse arrangement suffers from limited spatial resolution, sensitivity, and localization. For example, the ability to differentiate regions of activation is hindered because the channel density and spatial arrangement are directly related to the anatomical specificity.^23^ The downstream impacts of this vary. One possible scenario is that the channels miss a region entirely; another is that channels may read signal from multiple nearby regions, and without enough spatial diversity of channels to specify regional activation, the activation from multiple regions essentially gets averaged together. Either scenario prevents robust interpretation of brain behavior, whether in magnitude, regional specificity, or both.

In addition, sparse arrays are known to exhibit poor fNIRS signal reproducibility because of nonuniform spatial sensitivity.^24^ Though several works demonstrate that additional use of short-separation channels to the traditional sparse array improves data quality and sensitivity to cerebral hemodynamics by enabling regression of hemodynamics from superficial tissue from the long channel measurements,^25^^–^^27^ truly improved depth sensitivity, in addition, requires overlapping, high-density, multidistance channels at lengths that allow for cortical sensitivity. This type of layout can improve spatial resolution as well as related signal characteristics such as partial volume blurring, spatial and depth sensitivity, localization of brain response, and inter-subject consistency.^28^ These improvements are necessary to overcome limits of fNIRS’ ability to compare task-evoked response between conditions or between brain regions,^29^^,^^30^ for example, and in applications toward current important work in malnutrition in global contexts,^31^ brain disorders,^32^^,^^33^ surgery assistance,^34^^,^^35^ and brain-computer interfaces^36^ among others.

Recent advances in miniaturization of hardware components and fNIRS technology have made it possible to develop systems with high-density and overlapping, multidistance channels. This approach, high-density diffuse optical tomography (HD-DOT), was first introduced in 2007,^37^ and the first fiberless HD-DOT was introduced in 2016.^38^ The fiberless characteristic makes a system “wearable” and is important for approaching true ecological validity. HD-DOT attains high degrees of sensitivity, and, in fact, Eggebrecht et al.^39^ showed their system’s sensitivity approaches that of fMRI.

It has been demonstrated that localization can be improved through various methods to resolve high-density fNIRS signal depth.^31^^,^^40^^–^^43^ Currently, there are few commercially available HD-DOT systems^44^^–^^46^ as well as several systems developed in labs.^38^^,^^44^^,^^47^^,^^48^

As more fNIRS and DOT options become available, it will be necessary for users to be able to quantitatively compare systems to select an optimal setup for the needs of their application. A key drawback of using an HD system over a sparse system for a given regional coverage is the increased cost of resources such as materials—optodes, optode-to-cap attachments, control boards, other hardware—and computing processing required to efficiently process the larger amounts of data, especially in the case of whole-head arrays. When considering applications to resource-limited settings, for example, the priority of this characteristic increases. In addition, regardless of application, increasing the number and density of optode modules needed for the HD system especially in regions with hair incurs an increased setup and signal optimization time. The potential user needs to assess if the improved signal quality characteristics afforded by HD-DOT over traditional sparse fNIRS are necessary to achieve the goals set out in their investigation and justify the disadvantages that come from having a denser array. It may be that the goal is to simply capture activation in a broad field-of-view, compare task-evoked response, localize activation, perform connectivity analysis, or achieve localization consistency, all of which may have varying degrees of improvement from an HD array. It is critical, therefore, that the field provides a direct quantitative comparison of HD performance to that of traditional fNIRS arrays.

A relevant study by Shin et al.^40^ compared various fNIRS channel length combinations ranging from 15 to 35 mm and effectively compared HD with sparse fNIRS. The signal comparison was evaluated by classification accuracy, which demonstrated that the combination of multidistance and overlapping channels resulted in better classification accuracy than did a standard 30 mm grid layout. Although this supports moving toward overlapping HD arrays for the specific application, there remains a need to statistically compare other metrics of fNIRS measurements of functional activity.

To that end, to the best of our knowledge, there are two studies from Fishell et al.^31^ and Frijia et al.,^49^ which performed a direct comparison between a sparse and HD layout with matching field-of-views and concluded that HD arrays provide better localization of functional activity. The studies were in developing age populations, and the array comparisons were made between their entire HD probe and a subset of channels to form a sparse array from the same data collection.

Fishell et al.’s fiber-based HD system was composed of three channel lengths (13, 29, and 39 mm) and spanned the temporal and occipital regions; the “sparse” array measurements were compiled from the 29 mm channel data. The image results qualitatively showed that the HD-DOT layout provides greatly improved localization of functional activity at the subject and group level and demonstrated inter-subject localization consistency, though the results did not report a statistical comparison between the array results. In addition, the sparse sub-layout was not in a grid pattern nor did it include short-separation channels for superficial tissue regression, so it therefore did not best represent what is currently most commonly seen in sparse fNIRS systems.

Frijia et al.’s study used the wearable, fiberless GowerLabs’ Lumo components for their HD layout with multiple channel lengths (ranging from 10 to 45 mm) primarily over the superior temporal lobes; the “sparse” array uses only the data from a subset of nonoverlapping channels, the length of which is between 20 and 24.5 mm. Their HD results showed higher amplitude of hemodynamic response (HRF) compared with sparse results in both channel and image space. In addition, the SNR of their channel HRF was increased in HD measurements compared with sparse, and qualitatively the images showed improved and more consistent localization by the HD array. Although these results take the comparison of sparse and HD arrays a step further, neither array included short-separation channels for regression of superficial tissue hemodynamics. Statistical comparison between array measurements was not provided.

The agreement among these studies’ findings is encouraging and reinforces the potential for improved localization with HD fNIRS. Building on this foundation, our study provides complementary evidence by conducting a direct statistical comparison of sparse and HD fNIRS arrays. Furthermore, it extends prior work by examining array performance across different conditions that vary in cognitive load, as well as applying short-separation regression, offering deeper insights into optimal array design for specific applications.

Our work performs a direct comparison of measured word-color Stroop (WCS)-induced frontotemporal activation in 17 healthy adult subjects as detected by both a traditional grid-pattern, sparse fNIRS array and a hexagonal-pattern, overlapping and multidistance, high-density (HD) array to quantify the expected improvements afforded by the HD array. The WCS paradigm has been shown to elicit activation in the dorsolateral prefrontal cortex (dlPFC) due to the required response inhibition processing^50^^–^^57^ so our probes are designed to extend across the PFC. We have modeled a sparse optode layout after one of the most commonly used commercially available systems, ETG-4000 (Hitachi Medical Co., Tokyo, Japan) and our novel HD array is patterned after the NinjaNIRS^47^ layout and designed to have a field-of-view matching that of the sparse array. After standard signal pre-processing and image reconstruction, we generate concentration amplitude and t-statistics per subject for oxygenated and deoxygenated hemoglobin across WCS blocks for the channel data as well as brain surface vertices in image data. These metrics are both visualized and statistically compared at the group level. We find, in agreement with previous studies, that the HD array provides better localization than the sparse array. We also find that the HD array captures, on average, stronger signal than does the sparse array. The sparse array is suitable for detecting, though not localizing, presence of activity for the incongruent WCS but not for congruent WCS. Our resulting comparison of layout and paradigm conditions provides a useful reference for future fNIRS users to make an evidence-based evaluation of optimal probe design for their application given experimental needs and limitations.

## 2 Materials and Methods

### 2.1 Participants

Twenty-three healthy adult subjects were recruited from the Boston, Massachusetts, United States, area for this study via methods approved by the Boston University Charles River Campus Institutional Review Board (IRB Protocol 4502). Subjects gave written consent prior to the start of data collection. Subjects were not eligible for the study if they were outside the range of 18 to 89 years of age, had history of neurological trauma or neurological or psychiatric disorders, were currently taking psychoactive medications, were wearing a pacemaker or implantable cardioverter defibrillator, were wearing a deep brain stimulator, were wearing cochlear implants, had an uncorrected visual problem, or had a history of hearing problems. After subject exclusions due to technical errors (i.e., loss of signal transmission between devices during data collection) and poor data quality assessed during processing, 17 subjects remained, the data of which was used in analysis. Their demographics are as follows: mean age = 25.8 ± 4.3 years; 8 females, 9 males; 14 right-handed, 2 left-handed, 1 not reported; 10 White, 8 Asian, and 1 not reported.

### 2.2 fNIRS Measurements

#### 2.2.1 Optode arrays

**Fig. 1:**
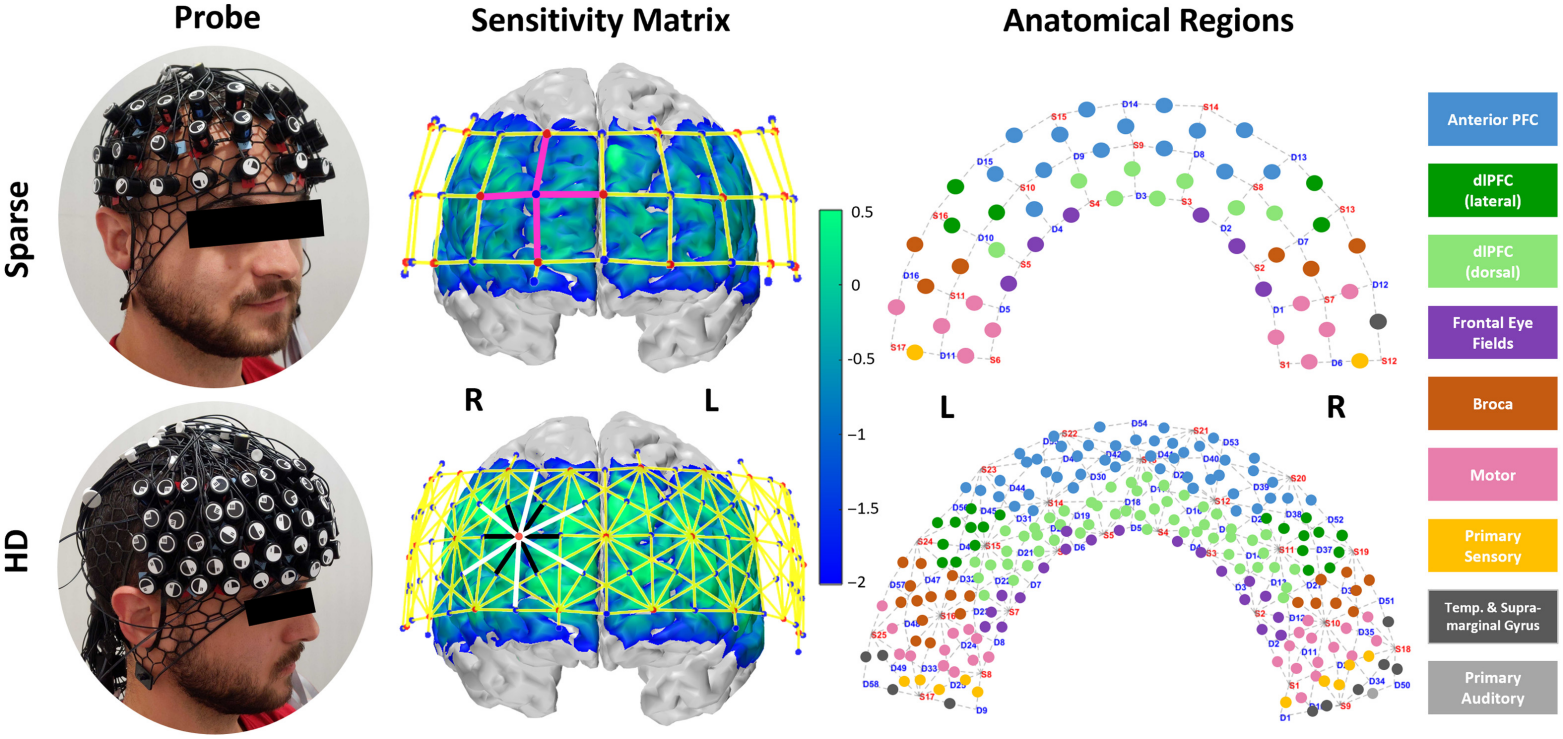
For each of the sparse and high-density (HD) probe designs, columns display physical appearance, sensitivity matrices via Monte-Carlo photon path modeling with probe overlay (red dots: sources, blue dots: detectors, pink lines: emphasize “grid” layout of sparse array’s 30 mm channels, black/white lines: emphasize “hexagonal” layout of HD array’s 19  mm/33  mm channels), and Brodmann areas underlying each channel. Sensitivity profile is on a log 10 scale; vertices with values >0.01 are not masked and not considered part of the relevant sensitivity profile.

We designed two optode layouts using the open-source AtlasViewer platform.^58^ They are openly accessible at Ref. 59. We refer to our “sparse” array as one which represents a traditional arrangement, depicted in the top row of Fig. 1. Its channels are in a grid pattern with lengths of 30 mm. The centered and bottom-most optode is anchored to FPz and the optodes at the left and right bottom corners are anchored to T7 and T8, respectively. This layout includes 17 sources and 16 detectors for 52 channels, with an additional 8 detectors at 8 mm separation (for a total of 24 detectors and 60 channels). Our multidistance, high-density probe layout, referred to as the “HD” array, is shown in the bottom row of Fig. 1. The HD array was designed such that its field-of-view is as close as possible to that of the sparse array, given pattern allowance. The centered optode on the bottom row is similarly anchored to FPz; the left and right corner optodes are just outside of the T7 and T8 landmarks with spring lengths of 2 mm to the landmarks. Following the hexagonal pattern recommended by von Lühmann et al., first nearest-neighbor (NN) channels are 8 mm, second NN channels are 19 mm, and third NN channels are 33 mm. There are 25 sources and 58 detectors which produce 112 19 mm channels and 94 33 mm channels, along with the additional 8 detectors at 8 mm length short separation channels (for a total of 66 detectors and 214 channels). The files for each of these arrays are available to download at Ref. 59.

To identify Brodmann regions underlying each channel, we employed AtlasViewer’s “project channels to surface” function to calculate MNI coordinates for each channel of our sparse and HD arrays. We then utilized the BioImage Suite Platform^60^ which maps the Colin27 brain surface (as used in AtlasViewer and therefore in creating our digital probe design) to the Talairach atlas surface and identifies Brodmann regions from the calculated MNI coordinates.^61^ In our case, not every channel was assigned a region from these steps because the coordinate was not located on the cortex in the BioImage platform. To complete the anatomical labeling of our probes as shown in the right-hand column of Fig. 1, we developed four steps. The first is spatial interpolation per array, meaning that if an unlabeled channel is surrounded by channels with uniform labeling, that label was applied. Next, we compare an overlay of sparse and HD channel labels; if one array’s unlabeled channel overlaps with or is surrounded by labeled channels from the other array it was assigned correspondingly. Thirdly, within each array we assigned still unlabeled channels with that of the mirrored channel in the other hemisphere. We acknowledge no individual brain, and thus the Colin27 template itself, is perfectly symmetric across hemispheres but at the group level symmetry can be assumed for the sake of region identification. Finally, for remaining unlabeled channels, we projected them to the cortex by applying incremental coordinate adjustments in the BioImage platform.

#### 2.2.2 Hardware

For both the sparse and HD layouts we used NIRSport2 optodes and systems (four cascaded NIRSport2 16×16 devices for HD and two for sparse) and Aurora data acquisition software (NIRx, Berlin, Germany). We used the dual-tip sources, which emit light at 760 and 850 nm, and silicone photodiode detectors. The sampling frequencies were 24.4 Hz for sparse and 17.5 Hz for HD. We manually optimized the spatial multiplexing of the HD array from its default settings to achieve its sampling frequency. We used fully customizable, conformable NinjaCap,^62^ 3D printed in three sizes (54, 56, 58 cm circumference) with NinjaFlex (Fenner Precision Polymers, Lititz, Pennsylvania, United States), a flexible thermoplastic polyurethane filament. Each cap included a reference marker for the Cz landmark. The open webbing design of the cap improves accessibility to maneuver hair to improve scalp coupling across diverse demographics. This cap structure also allows more heat to escape, thus increasing subject comfort.

### 2.3 Experimental Procedures and Paradigm

#### 2.3.1 Experimental procedure

For a given session, a subject first wore one array while performing the task, then after an ∼30-min break during which optodes were moved to the other cap, the subject repeated the task while wearing the other array. We randomized the order in which the arrays are worn to ensure roughly half the data was from subjects who first wore the sparse array and half from subjects who first wore the HD array.

To place the head caps, we first measured circumference, distance from inion (Iz) to nasion (Nz), and distance from ear-to-ear or LPA (T9) to RPA (T10). The cap for the selected measurement was placed to align both the Cz landmark and FPz optode on the cap to the subject’s head. Cz was measured as the intersection of the midpoint of a line connecting the Iz and Nz landmarks with the midpoint of a line connecting the LPA and RPA landmarks. FPz was measured from Iz as 10% of the total distance from Iz to Nz. Due to the nature of skull shape and size variety, cap placement according to one of these landmarks did not always converge to placement according to the other landmark. In cases where this was observed, alignment to FPz was prioritized because the array is focused on the frontal region. To reduce likelihood of hair falling between the optodes and scalp especially on the forehead and temple we first requested subjects look upward and set the frontal portion of the cap on first, then instructed them to look straight ahead and gently unfolded the cap over the rest of the head from front-to-back with one hand while lightly holding the front of the cap in place with the other. Scalp coupling optimization generally required more time and care for the HD array due to the increased number of optodes, which not only required more optodes be attended to, but in addition prevented easy displacing of hair from under one optode without accidentally pushing it under the next. Generally, moving hair was performed with a cotton-tipped applicator. To achieve the best signal quality, with the permission of the subject we sometimes applied clear ultrasound gel via the applicator. The optode was removed from the grommet, gel applied to the region as hair was pushed to the sides, and optode replaced; the gel aided to maintain hair position so that it did not move back under the optode. Although sometimes good scalp-coupling—as defined by the Aurora ‘green’ thresholds of covariance <2.5% and raw signal detection >3  mV—was achieved nearly immediately for most or all channels especially with bald or short hair, we would spend a maximum of ∼20  min to optimize the scalp-coupling before moving on. Room lighting was provided by halogen floor lamps rather than overhead fluorescent bulbs to avoid interference from ambient pulsed light. To prevent effects on frontal detectors from ambient signal emitted by the paradigm-presentation laptop, we placed a black plastic shower cap on the subjects’ head over the optodes.

The duration of sessions from the time of consent to completion was ∼2 to 2.5 h.

#### 2.3.2 Paradigm

**Fig. 2:**
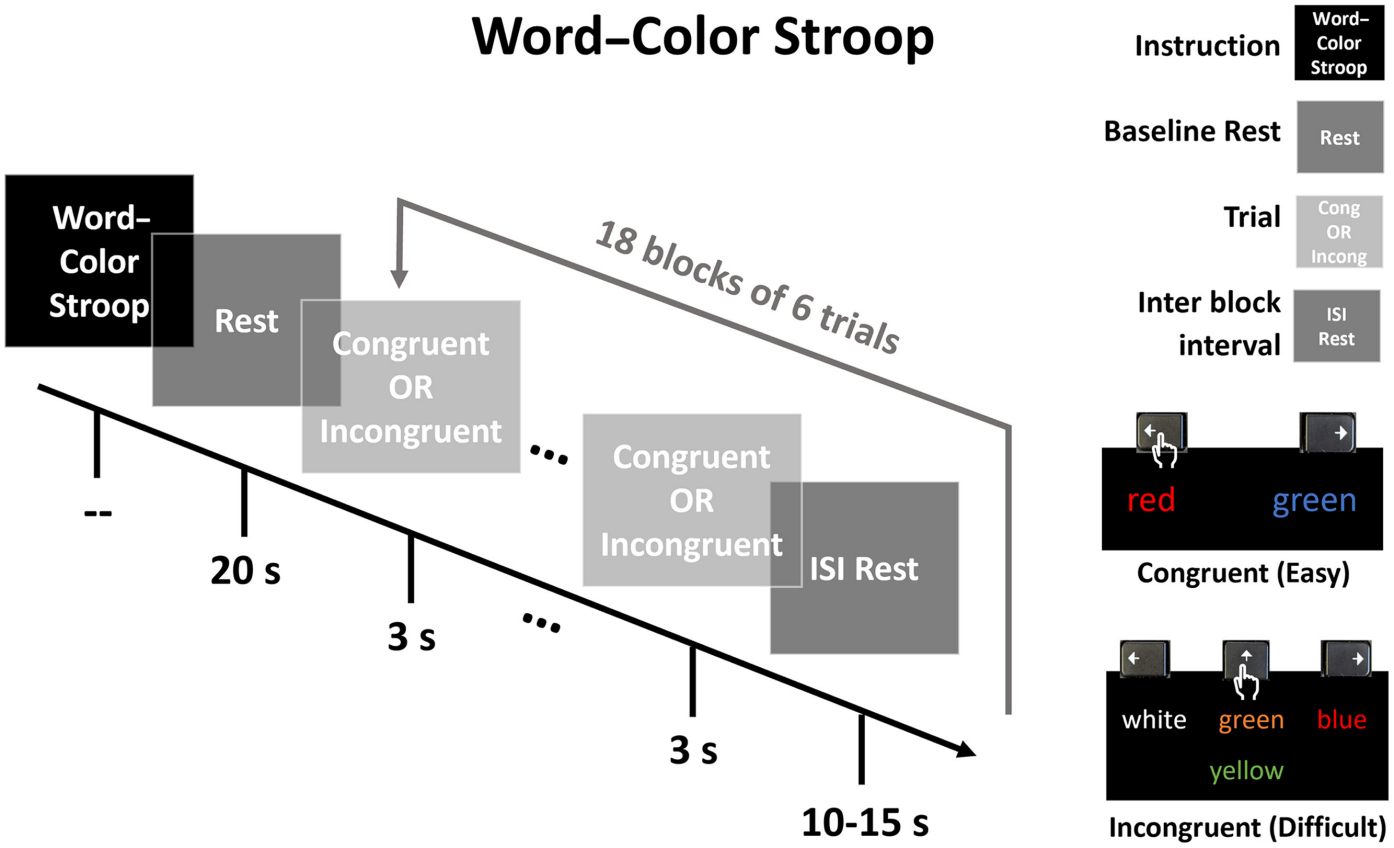
Word–color Stroop paradigm adapted from Jahani et al. After instruction and initial rest, 18 blocks of 6×3  s trials each were presented with a jittered inter-block interval (10 to 15 s). A given block consisted of either all congruent (easy) trials or all incongruent (difficult) trials. The lower-right legend demonstrates accurate user keyboard press for each condition. The order of blocks is randomized for a total of 9 blocks of each condition. The total run time is ∼11  min.

Our modified WCS task (Fig. 2) was based on previous work by Jahani et al.^51^ We compiled and presented it via PsychoPy^63^ with the full paradigm available on GitHub (https://github.com/andersonjessie/WordColorStroop). Our WCS had two conditions: an easier congruent task and a more difficult incongruent task. A trial of the congruent condition would display two words on a black background. Prior to a run, instructions for the congruent condition were provided on the screen and read aloud to the subject as follows: “For the following, two words will appear. Press either the Left Arrow or Right Arrow to indicate which word matches its font color. Press space to proceed to the practice.” The subject practiced twice with feedback, with additional practice if needed for a clearer understanding. The design of the congruent task ensured that per trial, one word was congruent to its font color and the other was incongruent to its font color. A trial of the incongruent condition would display four words on a black background. Instructions for incongruent were provided on the screen and read aloud to the subject as follows: “For the following, a word will appear in the middle and a set of three words will appear above it. Press the Left Arrow, Up Arrow, or Right Arrow to indicate which word of the top row (left, middle, and right, respectively) describes the font color of the word below. Press space to proceed to the practice.” Again, the subject practiced twice with feedback, with additional practice if needed for clearer understanding. Instructions and practices were repeated when the subject performed another run for the second array. The design of the incongruent task ensured all four words (the prompt word and three option words) were always incongruent to their own font color. In both conditions, the location of the correct response was randomized.

After completing instruction and practice, a run began with a 20 s rest, then 18×18  s blocks with interstimulus rest jittered between 10 and 15 s. There were 9 blocks of each condition, with the total block order randomized. Each block had 6 trials with a duration of 3 s per trial, and a given block was either all congruent or all incongruent trials with no feedback on accuracy provided to the subject. The total run time was ∼11  min.

### 2.4 Data Pre-Processing

We performed the following pre-processing steps in the open-source Homer3 platform.^64^ First, channels with raw signal levels less than 0.001 intensity units (for NIRSport2 data, V) or with a signal-to-noise ratio value (defined as raw signal mean divided by raw signal standard deviation, calculated per wavelength) less than 5 were pruned from further analysis (hmrPruneChannels). Intensity was converted to optical density (OD) (hmrR_Intensity2OD). We used the SplineSG motion correction function, which applies both spline-interpolation and Savitzky–Golay filtering to correct for baseline shifts and spikes, respectively (hmrR_MotionCorrectSplineSG),^65^ setting parameter p value for smoothing to the recommended 0.99 and using a 10 s frame size. A low-pass filter (hmrR_BandassFilt: Bandpass_Filter_OpticalDensity) of 0.5 Hz was used to remove high-frequency noise such as cardiac signal before OD was converted to oxygenated-, deoxygenated-, and total-hemoglobin concentrations (HbO, HbR, HbT, respectively) via the Beer-Lambert Law (hmrR_OD2Conc).^66^ Upon visual inspection of raw signal, OD, and concentration timeseries data, it was apparent that some of the slow motion artifacts in the temporal regions could not be fully corrected via available detection or correction functions, so we performed manual rejection of blocks which contained such artifacts. We then applied a general linear model (hmrR_GLM) to estimate the HRF from two seconds prior to block onset to 23 s after block onset. This captures 5 s after stimulus offset, at 18 s, to include the recovery period. In the GLM we used ordinary least squares and applied a consecutive sequence of Gaussian functions of 1 s width and step. For each long channel, we use the short-separation (8 mm) channel with the greatest correlation to regress the superficial tissue signal. The GLM included a polynomial drift correction of order 3.

If a subject had fewer than half the maximum blocks remaining in analysis due to manual block rejection (i.e., if they had four or fewer blocks for either condition), they were excluded from further analysis, which was the case for four subjects.

### 2.5 Image Reconstruction

To generate our sensitivity matrices per array, we performed forward modeling of photon migration via AtlasViewer’s “runMCXlab” function. This uses the MCXLAB package^67^ to perform a Monte Carlo simulation in which the random walk of photons between each source and detector is mapped to generate the sensitivity profiles.^68^^–^^70^ This represents the spatial sensitivity of each measurement channel to changes in cortical absorption. We chose to use the default parameters for photons launched (1e7) per optode and used wavelength-appropriate tissue properties for scalp, cerebrospinal fluid (CSF), gray matter, and white matter tissues.^27^ Each row of the sensitivity matrix represents the sensitivity profile of an individual channel. By summing over all channels, one can visualize the total sensitivity of the probe design as shown in the middle column of Fig. 1.

Image reconstruction was performed to project optical density in channel space to concentration on the cortical and scalp surfaces. To do this, the estimated HRF output for HbO and HbR from the GLM was first converted back to optical density. Channel space optical density measurements can be expressed as the product of the sensitivity matrix and the concentration changes on the cortical surface using the forward model:

y=Ax, (1)

where y is the channel space measurements in optical density, A is the sensitivity matrix and x is the changes in HbO and HbR on the cortical surface. To obtain these cortical measurements of HbO and HbR, we could solve the corresponding generalized inverse problem:

$$x=A^{-1}y.$$ (2)

Reconstructing images on both the brain and scalp simultaneously has been shown to improve image resolution and localization.^27^^,^^42^ Since the scalp is significantly more sensitive than the brain, spatially variant regularization is used to tune the reconstruction to the appropriate depth.^42^ This is done by rescaling A as A^ using the diagonal matrix L defined as:

$$\mathrm{diag}(L)=\sqrt{\mathrm{diag}(AA^{T})+\lambda_{\text{spatial}}},$$ (3)

$$\lambda_{\text{spatial}}=\alpha_{\text{spatial}}\;\max(\mathrm{diag}(AA^{T})).$$ (4)

Then,

A^=AL−1. (5)

The parameter $\alpha_{\text{spatial}}$ is used to control the reconstruction depth and is set to 0.001.

This inverse problem is ill-posed and underdetermined, however, so Tikhonov regularization was used to improve the estimation of x. The regularization parameter $\alpha_{\mathrm{meas}}$ was used to scale the measurement covariance such that, when combined with the spatially variant regularization, the inverse problem became:

x=L−1A^T(A^A^T+λI)−1y, (6)

λ=αmeas*max(diag(A^A^T)). (7)

The parameter $\alpha_{\mathrm{meas}}$ was used to smooth the image and was set to 0.001. We refer to this method as “brain and scalp” image reconstruction throughout.

To visualize a subject’s given set of vertices’ time course, we extracted the Homer GLM output HRF concentration data, generated the brain and scalp image for each second of HRF results, and plotted the mean of the vertices’ reconstructed image concentration over time.

### 2.6 Statistical Analysis

#### 2.6.1 Region of interest

**Fig. 3:**
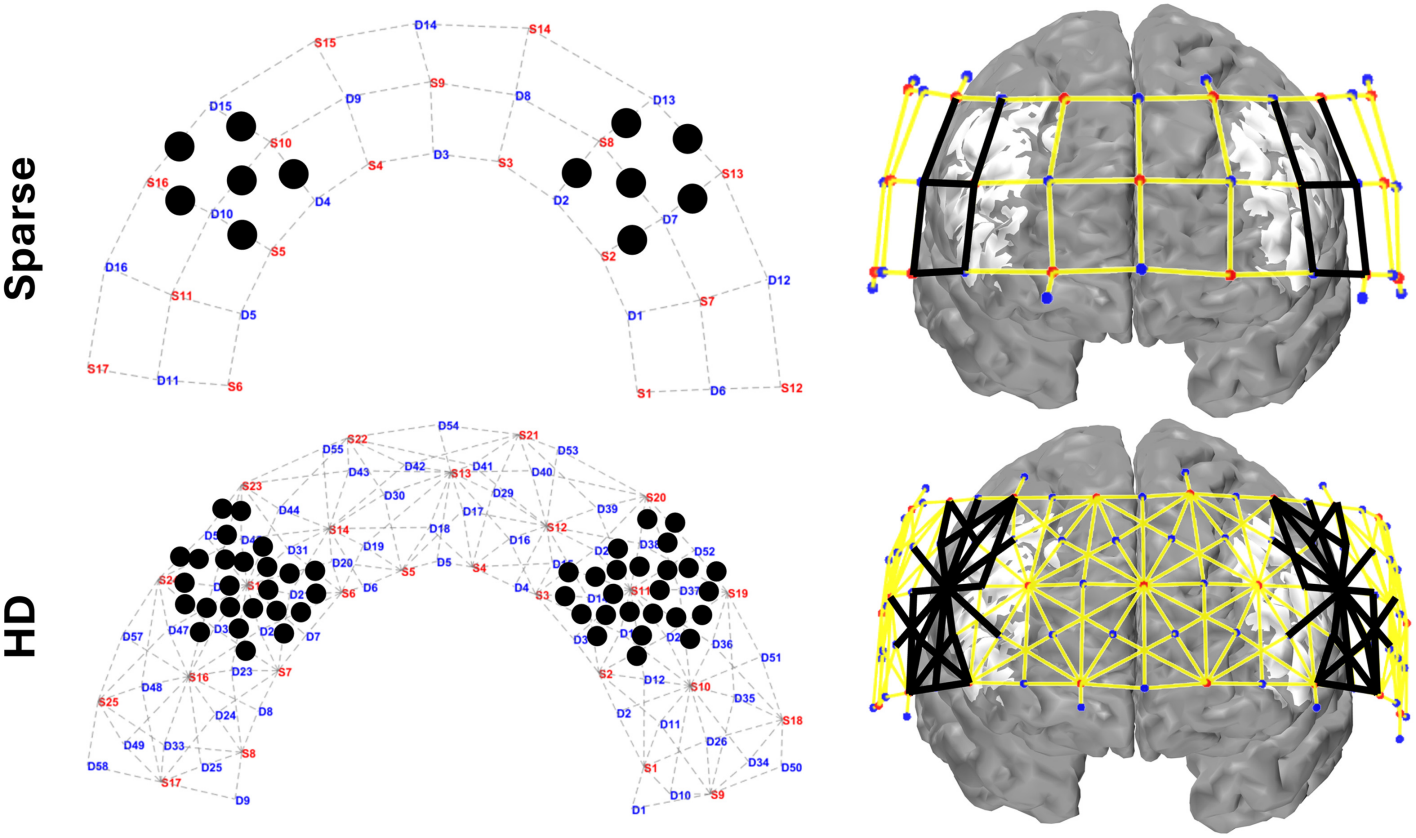
Channels and vertices selected in the region of interest for the sparse and HD array. On the left panel, black dots mark the center of each channel included in the ROIs. On the right panel, black lines indicate the channels included in the ROIs and the white (unshaded) region of the brain indicates the vertices included. Vertices for both arrays are chosen based on those sensitive to the HD ROI channels.

We selected the dlPFC as our region of interest (ROI) as that is where we expect the greatest activation to Stroop task based on previous studies.^54^ We included channels with this labeling in Fig. 1 and with a similar field of view, excluding the ones assigned as dlPFC (dorsal), which fall in the medial area. In selecting ROI channels, we preserved symmetry of channels selected within and between each array, as well as accounted for inter-subject brain variability, which led to including several channels not initially marked as in the dlPFC. (Sparse ROI channels included: 8 dlPFC, 3 Anterior PFC, 1 Broca. HD ROI channels included: 42 dlPFC, 4 Anterior PFC, 2 Broca, 1 Frontal Eye Fields.) The channels from each array selected per ROI appear in the left column of Fig. 3. Per ROI, there are 6×30  mm channels from the sparse array, 11×19  mm channels from the HD array, and 14×33  mm channels from the HD array. In selecting image space ROIs, because both the sparse and HD arrays have the same total vertices and near-matching placement, we selected exactly matching ROI vertices. We first identified the vertices whose sensitivity to the HD ROI channels was above the same 0.01 threshold used in visualizing the sensitivity matrices. We then averaged the sensitivity value of those vertices per ROI, and kept the vertices per ROI that had a sensitivity value greater than the ROI average as shown in the right column of Fig. 3.

#### 2.6.2 Array comparison statistics

We analyzed the processed concentration timeseries data for HbO and HbR, and for both congruent and incongruent conditions. For each block at a given channel of an individual subject, we calculated a delta concentration by subtracting the mean of the concentration time course from −2 to 0 s prior to block onset from the mean of the concentration time course between 7 and 18 s after the block onset. The mean delta concentration across blocks was divided by the standard error across blocks to produce a t-statistic particular to that concentration, WCS condition, channel, fNIRS array, and subject. For visualization purposes, group-level concentration means and standard error from across subjects’ own block-averages was used in calculating group-level t-statistics per channel.

To compare sparse and HD HbO results, we selected from each subject the channel per left and right ROI that had the highest t-statistic. For HbR results, we selected based on lowest t-statistic with the evidenced expectation that HbR decreases when HbO increases for typical brain activation.^71^ We performed a two-tailed paired Student’s t-test on the concentration data from the selected channels to statistically compare array performance.

To analyze in image space, we first reconstructed the image of each block based on the peak minus baseline values per channel previously calculated. We then used similar methodology to analyze the image data as used to analyze channel data, applying it to the mean of the 25 image vertices with highest t-statistics.

## 3 Results

### 3.1 Data Quality and Retention

**Fig. 4:**
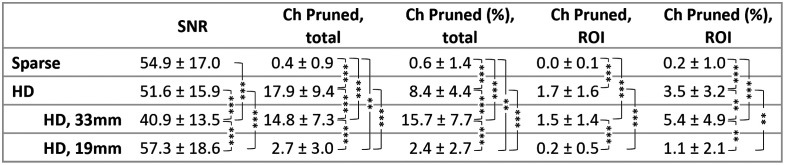
Channel signal quality metrics are provided in terms of mean and standard deviation across subjects. * for p≤0.05, ** for p≤0.01, *** for p≤0.001 by two-tailed paired t-test.

Channel quality results are presented in Fig. 4. Here we focus on the report of all length HD channels. By two-tailed paired Student’s t-test, there was no significant difference between the SNR of the sparse array channels and the SNR of the HD Array channels (p=0.364). There was, however, a significant difference in the number and percentage of channels pruned from each cap (p≤0.001 for both). The differences were also significant when looking specifically within the ROIs (p≤0.001 for both). The number of channels kept (pruned) in the analysis were, on average, 196.1±9.4 (17.9±9.4) HD channels and 59.6±0.9 (0.4±0.9) sparse channels across the entire array; within our ROIs there were on average 48.3±1.6 HD channels and 12.0±0.1 sparse channels included in the analysis. There was no significant difference between the number of blocks available for a given WCS task when the subject wore the sparse array versus when they wore the HD array (p=0.361 for incongruent, p=0.206 for congruent by two-tailed paired Student’s t-test). There was also no significant difference between the number of blocks available when wearing a given array for the incongruent versus congruent task (p=0.311 for the sparse array, p=0.361 for the HD array via two-tailed paired Student’s t-test).

### 3.2 Performance Metrics

Table 1 provides the group accuracy and response times (RT) per array and WCS condition, based on data from 16 subjects whose performance metrics were available for analysis. Between arrays, there was no significant difference in the accuracy or RT (p=0.545 for congruent accuracy, p=0.937 for incongruent accuracy, p=0.398 for congruent RT, p=0.990 for incongruent RT, two-tailed paired Student’s t-test). Accuracy did not significantly differ between congruent and incongruent tasks (p=0.051 for sparse array, p=0.069 for HD array). Response times differed significantly between the conditions, with slower responses in the incongruent task (p≤0.001 for both arrays’ RT, two-tailed paired Student’s t-test).

**Table 1:** Performance metrics are provided as average across subjects’ trial averages. p≤0.001 between items with superscript A (condition response times for sparse array), and between items with superscript B (condition response times for HD array) via two-tailed paired Student’s t-test.

	Accuracy (%)		Response Time (s)	
	Congruent	Incongruent	Congruent	Incongruent
Sparse	97.8 ± 4.7	86.8 ± 21.9	1.05 ± 0.27^A^	1.57 ± 0.24^A^
HD	97.1 ± 8.3	87.2 ± 18.5	1.08 ± 0.25^B^	1.57 ± 0.19^B^

### 3.3 Functional Activation

**Fig. 5:**
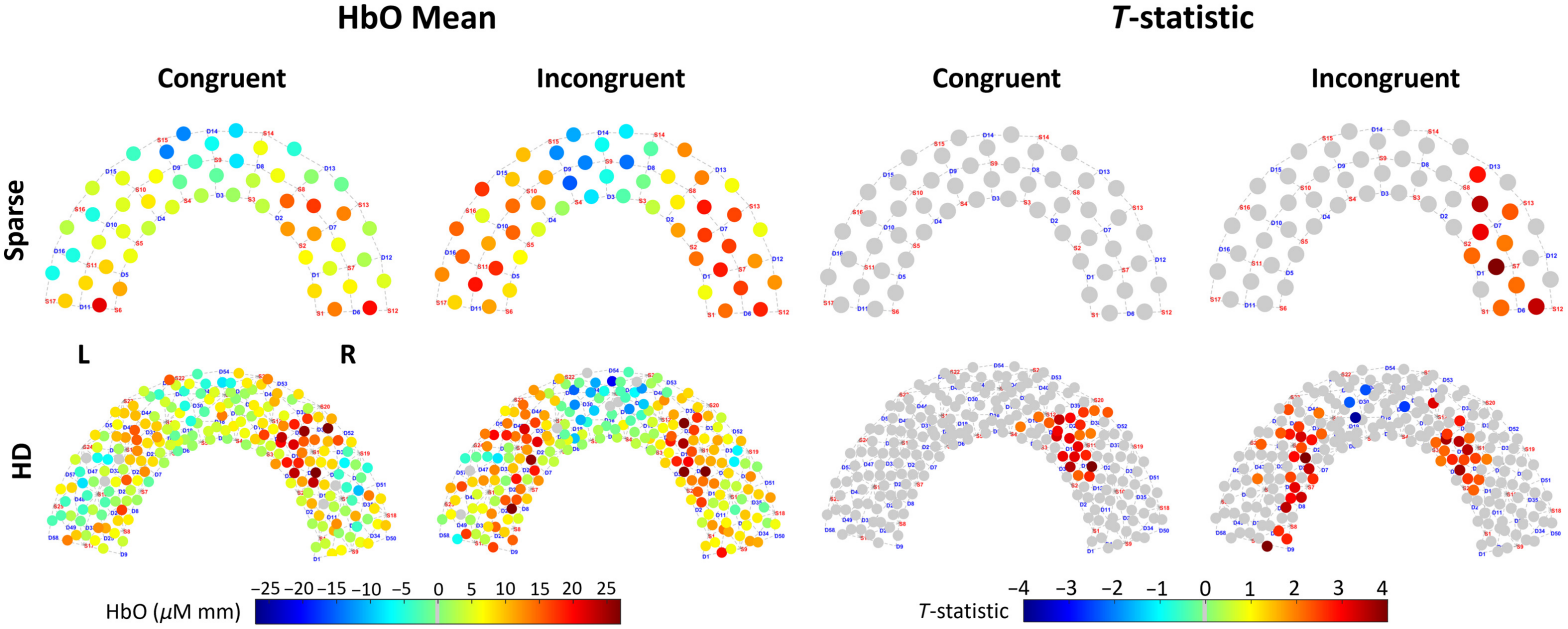
Channel space brain response recorded by sparse and HD arrays during WCS, from Superior view. “HbO Mean”: Group-average hemodynamic response (HbO) for each channel, averaged across 7 to 18 s of the blocks for each condition. “T-statistic”: t-statistic of each channel across subjects is plotted. Gray channels have a p-value greater than critical p-value calculated from a cluster permutation, varies per plot.

**Fig. 6:**
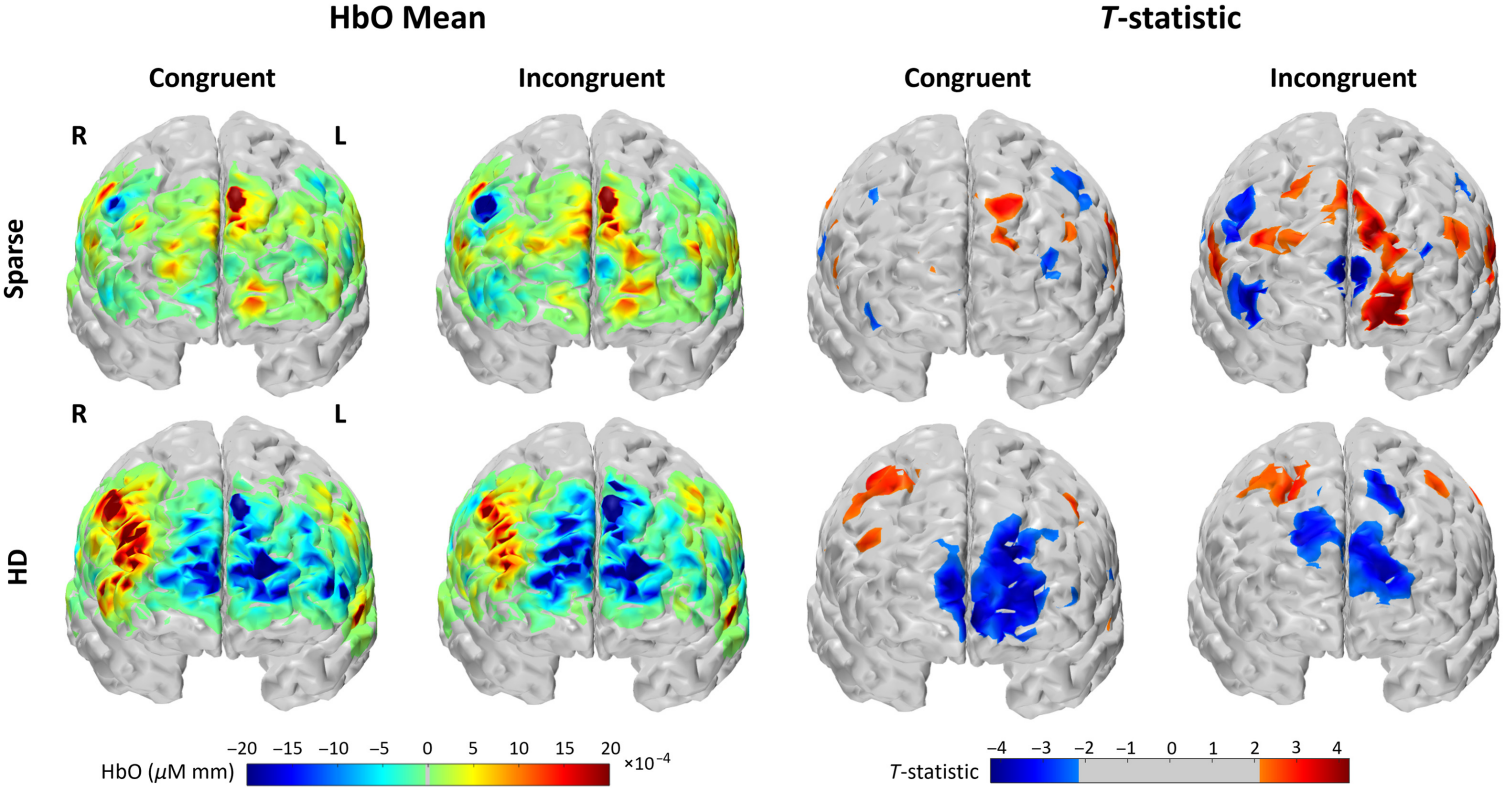
Brain and scalp image space brain response recorded by sparse and HD arrays during WCS, from Anterior view. “HbO Mean”: Group-average hemodynamic response (HbO) for each condition. “T-statistic”: t-statistic of each vertex across subjects is plotted. Color-scale is gray for absolute values less than t-critical = 2.12 as calculated for 17 subjects.

For each WCS condition and array, group-averaged mean HbO and t-statistic are shown in channel space in Fig. 5. The reconstructed image results for group-averaged HbO mean and t-statistics are shown in Fig. 6. Functional activation was present in the lateral regions for the HD array for both conditions. Functional deactivation was also present in the medial PFC for the HD array, seemingly more strongly during incongruent WCS than during congruent WCS when looking at channel space. The pattern of activation in the sparse image is difficult to distinguish and for the most part opposite of what is expected according to the channel group results. Due to the variation of channels pruned per subject, not all the group channel calculations had the same number of subjects whose data were contributing. On average, HD channels’ group data had 15.6±2.4 subjects per channel, and sparse channels’ group data had 16.9±0.4 subjects per channel. For visual and comparative continuity across caps in image space, the maximum n of 17 (with two-tailed α=0.05) was used to calculate the critical t-statistic value of 2.1 for group data. We mask vertices with t-statistics below this threshold by visualizing them as gray, as seen in Fig. 6. In channel space, a cluster-based permutation test with a radius of 33 mm, alpha of 0.05, and 5000 permutations was applied to each array and group result. This affects the critical t-statistic value for each group result, below which the channels are masked by visualizing them as gray, as seen in Fig. 5.

The Supplementary Material includes similar plots for HbR in channel and brain and scalp image space (Figs. S1 and S2), and both HbO and HbR group-average channel timeseries (Fig. S3). We have also included all individual subject HbO mean and statistical results for incongruent WCS in channel and image space in Figs. S4–S7.

### 3.4 Statistical Comparison

**Fig. 7:**
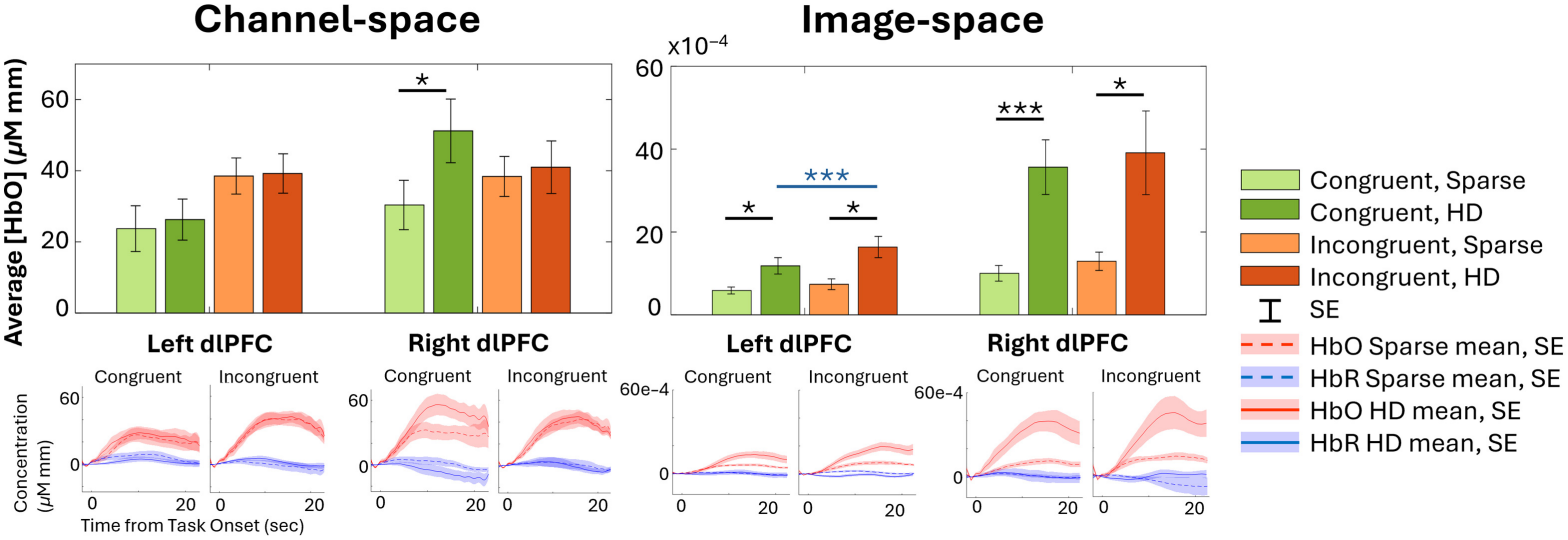
From within the ROIs, group-averaged HbO from channels or vertex clusters with maximum t-statistics are presented in both channel and brain and scalp image space for each array and WCS conditions. Asterisks indicate * for p≤0.05, *** for p≤0.001 for two-tailed paired Student’s t-test between arrays (black) and conditions (blue). The subjects’ selected channel or averaged 25 vertices’ concentration time courses are averaged for the timeseries plots. Numerical average, standard error, and two-tailed paired Student’s t-test values are available in Table S1 in the Supplementary Material.

From the ROIs, HbO concentration from channels or vertices with the maximum HbO t-statistics per subject were compared between arrays as in Fig. 7. The corresponding average time course from each channel or vertices selected for a given condition, array, and ROI across subjects is shown underneath the bars with which it is associated. (The results for HbR data are in Fig. S8 in the Supplementary Material, and values for both Figs. 7 and S8 are provided in Table S1). Of the HD channels selected from each subjects’ ROI for its maximum HbO t-statistic during incongruent WCS, 8 out of the 17 were 19 mm. During congruent WCS, 8 of 17 channels selected in the left ROI and 5 of 17 in the right ROI were 19 mm. Further comparison of channel concentration when selecting from only the 33 mm channels or from only the 19 mm channels of the HD array is available in Fig. S9 and Table S2 in the Supplementary Material.

The channel ROI data from the HD array was significantly greater in magnitude than that of the sparse array in the right ROI for both HbO and HbR congruent WCS data (right congruent channel HbO p=0.026, right congruent channel HbR p=0.023). In both channel and image space, the HD array consistently provided a greater average concentration amplitude than did the sparse array for both HbO and HbR, and per WCS condition. The HbO image space results in both the left and right ROIs and for both WCS conditions were significantly greater from the HD array than from the sparse array (left congruent HbO p=0.017, right congruent HbO p≤0.001, left incongruent HbO p=0.015, right incongruent HbO p=0.019); the HbR image space results in the right ROI during both WCS conditions were also significantly greater in amplitude from the HD array than from the sparse array (right congruent HbR p=0.001, right incongruent HbR p=0.021). All p-values are provided in Table S1 in the Supplementary Material.

As a whole, in both channel and image space, the HbO statistics from the incongruent condition were greater than those from the congruent condition, with the exception of the right ROI for HD in channel space. The difference was significant in the left ROI for the HD array HbO data in image-space (p≤0.001).

## 4 Discussion

Though the prevalence of fiberless HD-DOT systems is increasing,^41^^,^^72^^,^^73^ there has been a lack of adequate characterization of the expected improvement of data obtained by such arrays over the commonly available traditional, grid-sparse fNIRS array. To our knowledge, this is the first study to perform a direct statistical comparison of two such arrays in a group of healthy adult subjects, providing statistical analysis in both channel and image space, including short-separation channels in the arrays to perform superficial tissue regression accordingly, and offering comparison for similar task conditions with differing cognitive load.

### 4.1 Channel and Image Statistics Array Comparison

Our analysis quantitatively compared the strength and consistency of signals detected from each array. Across all comparisons of HD to sparse data (in both channel and image space, in both the left and right dlPFC, during both WCS conditions, for both HbO and HbR), the group-averaged concentration of selected channels and vertices shows that the HD array can more consistently capture the strength of activation than the sparse array (see Fig. 7 and Fig. S8 in the Supplementary Material).

Interestingly, the 19 mm channels comprised approximately half of the HD channels selected from each subjects’ ROI for its maximum HbO t-statistic during incongruent WCS, as well as half of the selected channels for congruent WCS in the left ROI. We anticipated the 33 mm channels would yield signal with a higher t-statistics than the 19 mm channels due to higher brain sensitivity. Previous studies investigated the brain sensitivity of source-detector separations (SDS) ranging from 20 to 45 mm and found that for every 10 mm increase in SDS within this range, sensitivity to gray matter increased by ∼4%. In addition, the findings indicate that a minimum SDS of 25 mm is required to achieve relative sensitivity greater than 1% at a depth of 11.2 mm into the brain.^74^ However, our data suggests that 19 mm channels may perform comparatively well to 33 mm channels for measuring dlPFC in our healthy adult population, based on the t-statistic metric. For a possible explanation as to why the t-statistic is higher, we look at Fig. 4’s report that the average raw signal SNR of un-pruned 19 mm channels (57.3±18.6) was significantly higher than that of the un-pruned 33 mm channels (40.9±13.5). In addition, looking further into the standard error of HbO concentration across blocks, we found that the average SE of the 33 mm channels selected for maximum t-statistic was consistently higher than that of the 19 mm channels selected for maximum t-statistic (during congruent WCS: in the left ROI, 33 mm SE is 1.13±0.24 vs. the 19 mm SE is 0.99±0.18; in the right ROI, 33 mm SE is 1.76±0.27 vs. the 19 mm SE is 1.32±0.25 in right ROI. During incongruent WCS: in the left ROI, 33 mm SE is 1.09±0.16 vs. the 19 mm SE is 0.98±0.11; in the right ROI, 33 mm SE is 1.37±0.24 vs. the 19 mm SE is 1.11±0.22 in right ROI).

Our observation of the 19 mm’s surprisingly good performance inspired the re-running of the channel space statistical comparison, altered to select an HD channel from either only the ROI’s 19 mm channels, or only the ROI’s 33 mm channels (see Fig. S9 in the Supplementary Material). We recall that per ROI, there are 6×30  mm channels from the sparse array, 11×19  mm channels from the HD array, and 14×33  mm channels from the HD array. We found that compared with sparse data, the option of selecting from both 19 and 33 mm HD array channels and the option of selecting only from 33 mm HD array channels consistently yielded greater average concentration (i.e., higher average HbO or lower average HbR). Looking just at the HD array channel option, we observe that in some cases selecting channels from only among the 33 mm channels provided greater concentration than choosing from both 19 and 33 mm channels. In light of this, to provide explanation as to why some 19 mm channels get selected for greatest t-statistic when given the option to select from both 19 and 33 mm, we again note that the SE of the 19 mm channels was lower than the 33 mm channels which can lead to their having a higher t-statistic even if they have a lower concentration. Selecting from only 33 mm channels consistently provided a higher average maximum concentration than selecting from only 19 mm channels, which aligns with our expectations. It is not entirely clear if this may have occurred due to having more 33 mm channels available than 19 mm channels, or from improved signal from the 33 mm channels.

We initially also had different expectations for the image space results—specifically, that the HbO pattern from both arrays’ measurements would be similar to that of the channel space statistical comparison in Fig. 7, where there is dlPFC activation and mFPC deactivation for both tasks, more so during incongruent WCS. Although the HD array resulting pattern of activation aligned with the channel space results, the sparse array images are almost opposite, wherein there is more activation in mPFC and more deactivation in dlPFC. Because the HD image results align with the foundational assumption that activation patterns in image space should reflect those of channel space, and due to its improved sensitivity profile over that of the sparse array (Fig. 1), we therefore assume the HD image results are more reliable than the sparse array image results. We applied other variations for image reconstruction to assess if there is a method that provides appropriate results for both the sparse and HD image results, described as follows.

We explored a method for pushing physiological and measurement noise to the scalp surface while isolating the cortical activity to the brain surface; Gao et al. developed a method that uses spatial basis functions modeled as Gaussian kernels with σ=5  mm for the brain and σ=20  mm for the scalp.^27^ This has the advantage of reducing the degrees of freedom of the model and smoothing the resulting images. When we implemented this method, using the recommended parameters α=0.01 and β=0.1 in the combined spatially variant and Tikhonov regularization in the sensitivity matrix inversion calculation,^75^ it seemed to appropriately handle the HD array data. However, we saw an even more dramatic reduction of activation in the sparse array due to its lack of overlapping channels. This suggests that the traditional sparse array is ill-posed by nature for reconstructing an appropriate image that separates brain and scalp data.

We suspect that the sparse array is better represented by not including scalp surface via spatially variant regularization when performing image reconstruction, and we have therefore generated such group result images in Fig. S10 (Supplementary Material) for the sparse array, which we refer to as “brain only” image reconstruction. In doing so, we observe in the images and time course plots that while the HbO results appear more aligned with what is expected, there is strong cross-talk between the HbR and HbO data, which is not present in the regularized data. This demonstrates that the brain only method for reconstructing sparse data is also unfit. Therefore, for the purpose of directly comparing HD and sparse data, in our presented image results we chose to apply the same method to both arrays and include that in analysis in Fig. 7. Because our analysis selects the top vertices based on the HbO increase in the ROIs (and HbR decrease in the ROIs as shown in the Supplementary Material) we can still compare potentially relevant results, but we argue that it may be inappropriate to assess sparse image results as a whole.

For both arrays, it is possible that our ability to perform a more robust statistical comparison of image space results was hindered by the limitation that, like many other fNIRS studies, we did not have subject-specific MRIs to support more accurate probe localization. Rather, we used the AtlasViewer-provided Colin27 head atlas across all subjects.

### 4.2 Capturing Different Cognitive Load

This study allowed us to observe each array’s comparative performance for different cognitive loads. The Stroop effect was clearly demonstrated in our data by the increased activation during incongruent task as compared with congruent in both caps (activation pattern is discussed further in Sec. 4.3), with the exception of the HD channel data in the right ROI, and performance metrics were comparable to what was expected based on previous literature,^53^^,^^76^ so we are confident that our study setup generated desired brain activity. The performance metrics’ lack of significant difference in accuracy or response time between sparse and HD array verifies comparability of the WCS brain response between arrays. The presence of significantly longer response time during incongruent WCS than during congruent WCS while maintaining accuracy, both when wearing sparse and HD array, suggests and supports that there is a higher cognitive load when performing incongruent WCS (Table 1). This is expected since the congruent condition does not require interference resolution and response inhibition to identify the correct response, which supplies noninterfering information.

Although it was unexpected that, in HD channel space, average HbO was greater in the right ROI during congruent than incongruent WCS, other studies have found the left PFC to be more involved than the right in the Stroop effect.^77^ This emphasizes the greater importance of the left ROI demonstrating the anticipated greater activation during incongruent than congruent WCS, which both arrays demonstrate in channel and image space. Although this might indicate either array is suitable to find difference between task-specific activation in the most relevant ROI, looking at the activation patterns of each condition independently yields a more nuanced perspective. In channel space for the incongruent task, both arrays captured the presence of activation as seen in the statistical visualization of Fig. 5, to varying degrees of localization discussed further in Sec. 4.3. However, there was a markedly different ability between the arrays to capture activation during the congruent task. The HD array still captured robust activation patterns in channel space; by contrast the sparse array results have no channels at all that detect significant activation at the group level. Looking at image results in Fig. 6, the sparse array is similarly devoid of activation in the ROIs. For both tasks, though the channel comparisons statistics in Fig. 7 would suggest HD does not strongly outperform sparse except in the right ROI for congruent WCS, the image space comparisons suggest otherwise. In addition, the visualization in Fig. 6 clearly indicates the HD array consolidates, or localizes, activation for both tasks.

This has implications for future array choice which should depend on a given paradigm’s difficulty or expected magnitude of activation, and intention to analyze results in channel or image space. It is worth noting that most of our subjects have similarly high educational attainment, and the age range is limited which may skew the data. Future work might implement a similar comparative method with other tasks and conditions and across other populations to build a bank of array detection characterizations. This could enable better-informed selection of paradigm pairing with selected array density and location, whether for research, clinical use, or other purposes. In addition, due to the importance of differentiating subject-specific activity between conditions^78^ or condition-specific activity between subjects,^3^^,^^7^^,^^52^ more work could look at other condition-specific metrics, such as signal latency, integral value, or centroid value.^79^^,^^80^

### 4.3 Localization Comparison

The pattern of HbO activation during WCS as detected by both arrays in channel space and by HD array in image space matched that of previous fNIRS and fMRI findings specific to WCS^50^^–^^52^^,^^54^^,^^76^—that is, lateral activation and medial deactivation, more strongly in the incongruent condition than in the congruent condition (Figs. 5 and 6). This medial deactivation was observed in both channel and HD image space for the incongruent task. For the congruent task by HD array, however, it appeared only after image reconstruction, that is, in image space but not in channel space. Although this may indicate differences in superficial physiology between task conditions—signals that are attenuated during the image reconstruction step—we do not draw strong conclusions, as our relatively small sample size limits the reliability of condition-level contrasts. Although prior work (e.g., Chen et al.) has shown associations between fNIRS global signal and vigilance,^81^ their study did not involve task-based comparisons and thus does not support a condition-specific explanation in our case. We therefore interpret this pattern with caution and highlight it as an open question for future investigation. From the visualization of both arrays’ concentration data in channel and HD array in image space (Figs. 5 and 6) as well as the statistical comparison in channel space (Fig. 7), the incongruent task seemed to elicit a more uniformly bilateral response than the congruent task for which there is more activation present in the right ROI than the left. This hemispherical activation difference for congruent WCS was not observed in most other studies.^50^^,^^53^^,^^54^ However, it is known that the left dlPFC is implicated in interference processes like that induced by Stroop effect,^56^^,^^57^ so it follows that the difference of activation between congruent and incongruent tasks is greater in the left than right dlPFC which was true of our data as well (Fig. 7). Though we did not expect the lateral difference for congruent data, there is another published work of healthy adults which also found less activation in the left PFC than the right PFC for their nonincongruent task.^55^

Looking at the visualized regions of significance-thresholded activity (Figs. 5 and 6), especially for the higher-cognitive load incongruent task, it becomes clear that the HD array better captures and localizes the activation in both channel and image space. This validates the Monte Carlo simulated photon migration results which showed improved sensitivity in the HD array (Fig. 1). This also supports previous literature naming the same advantage in infant populations and without applying short-separation regression to all the data.^31^^,^^49^ This is most dramatically illustrated in the reconstructed image data (Fig. 6), for which the active region detected by HD is continuous and contains a relatively centered focus of higher activity. By contrast, the active region detected by sparse is discontinuous and lacks a centered focus of activity. Our study, therefore, translates some of the existing findings to the 3 cm grid array which is commonly available and in use and to an adult population, better enabling comparison of the systems and for broader application.

### 4.4 Signal Quality and Data Collection

Since our comparison occurred over the PFC, the probe designs did not cover as much hair as they would have for other regions such as parietal or occipital. This enabled us to perform the study without the need to assess how the presence of hair might affect the layouts differently, providing more of an ideal baseline comparison between the two. The left and right extremities of the arrays, though, do extend into hair regions especially on the superior boundaries of the cap, and we would predict that the HD array signal is more impacted by hair. Surprisingly, we saw that the SNR of the sparse and HD array were not significantly different for nonpruned channels (p=0.364), which demonstrates promise for comparable signal quality. However, the significantly greater percentage and number of channels pruned for low SNR and low raw intensity from the HD array than for sparse point to increased difficulty in achieving optimized signal via the HD array. Even so, we note that on average we still had over three times as many channels available from the HD array as compared with sparse; within our ROI we had over four times as many. On average, we retained 91.6% of channels in the HD array which is higher than the channel retention for previous HD studies.^31^ The HD pruned channels were mainly located in the superior portions of the array so we conclude the increased proportion of pruned channels from the HD array was due to presence of hair (see Fig. S12 in the Supplementary Material). Future work may characterize these metric comparisons in other regions of the head where there is more hair and hopefully elaborate on methods to reduce the percentage of HD channels pruned for SNR and low signal. We do recommend that users of fNIRS systems follow our described methods of cap placement and scalp coupling to enhance signal quality and use of an open-webbing headcap design such as the ninjaCap^62^ used in this study, in combination with other documented techniques,^82^^–^^86^ and look forward to further developments of user techniques or technologies to ensure good signal quality optimization across demographics. Anecdotally, we can speak to our subjects having a range of hair color, shape, and texture, as well as a range of skin tone, thus verifying the applicability of the findings across various racial demographics. However, we recognize the absence in our study and suggest future studies to regularly collect metrics of hair color, shape, and texture as well as skin melanin pigmentation metrics to continue documenting and improving fNIRS ability to accommodate for a full range of demographics.^86^

A challenge we faced was that the number of optodes we had available allowed for only one array to be assembled at a given time. Subjects provided their estimated head circumference ahead of the session, and therefore we could populate optodes on the NinjaCap for either HD or sparse for the estimated size. Unfortunately, subjects did not always provide an accurate head measurement as determined once we measured after consenting (differences were no larger than 2 cm). Due to time constraints for the full session duration, we chose to proceed with the pre-assembled cap size; when moving optodes to the other layout for the second run of the session we used the same cap size for per-subject size consistency. If a cap was too large for the subject’s head it may loosen the optode contact with the subjects’ head, which we accounted for by creating a fold in the occipital region (similar to a sewing dart) and securing with a clip. Although using caps of incorrect head size would not affect pairwise comparison of data, it is likely to, in small ways, have affected localization of group results.^87^ We recommend future work to navigate a challenge like this by a variety of methods, such as pre-study head measurements, digitizing probe localization after cap placement, using a size-adjustable cap design, or simply populating multiple cap sizes if the resources are available.

To perform a direct comparison of our HD array to the sparse grid array, we were constrained to using two separate printed caps and populating them separately due to nonoverlapping optode locations. Though this potentially introduced variability in cap placement per subject, our pair-wise analytical methods overcome this by selecting one channel or vertex per ROI with greatest t-statistic. In addition, any effect of variability in per-subject cap placement in group results would not be any greater than effects due to inter-subject cap placement variability.

## 5 Conclusion

As fNIRS development and research progresses toward whole-head HD systems, this work’s characterization in the PFC of the improvement our HD layout affords over that of the traditional sparse array may apply across the whole-head. Several key implications of sparse and HD array use emerged from our study, which can inform future selection of array and paradigm to best elicit and capture functional activity. Comparison of congruent task results suggests that the sparse array is inadequate for capturing brain activity when used for tasks that have a lower cognitive load in healthy adult subjects; conversely, our HD array is sufficient for capturing such brain activity. For both WCS conditions, localization is better achieved with the HD array and image reconstruction can be more appropriately performed with the HD array. If localization is not of high importance and analysis of image space results is not necessary, our incongruent results indicate that either array can be sufficient to capture the presence of activation when designed with continuous coverage over the ROI. The WCS task is relevant for many studies in psychiatric well-being and other cognitive applications; thus, these results can hopefully translate directly to inform future studies using WCS and related Stroop or response-inhibition paradigms. We hope our methods and findings offer a foundation on which ongoing fNIRS array comparisons, expansions, and applications may build.

## Supplementary Material

10.1117/1.NPh.12.3.035010.s01

## Data Availability

Both the sparse and HD frontotemporal array designs are openly accessible at https://openfnirs.org/hardware/ninjacap/ in file formats of .SD and .SNIRF for use with the AtlasViewer and Homer3 platforms, and in three circumference size files (54, 56, 58 cm) in .stl and Cura file formats for 3D printing. Code to run the word–color Stroop paradigm in PsychoPy is publicly accessible at https://github.com/andersonjessie/WordColorStroop. De-identified recorded fNIRS and task performance data are available in separate datasets at openneuro.org; for Sparse data: https://openneuro.org/datasets/ds006459, for HD data: https://openneuro.org/datasets/ds006460.
