# Supplementary material for: High-density multidistance fNIRS enhances detection of brain activity during a word-color Stroop task
Source: Neurophotonics. 2025 Sep 2;12(3):035010. doi: 10.1117/1.NPh.12.3.035010 (PMC12412631; doi:10.1117/1.NPh.12.3.035010)
Supplement: Supplementary file 1 [file NPh_012_035010_SD001.pdf]

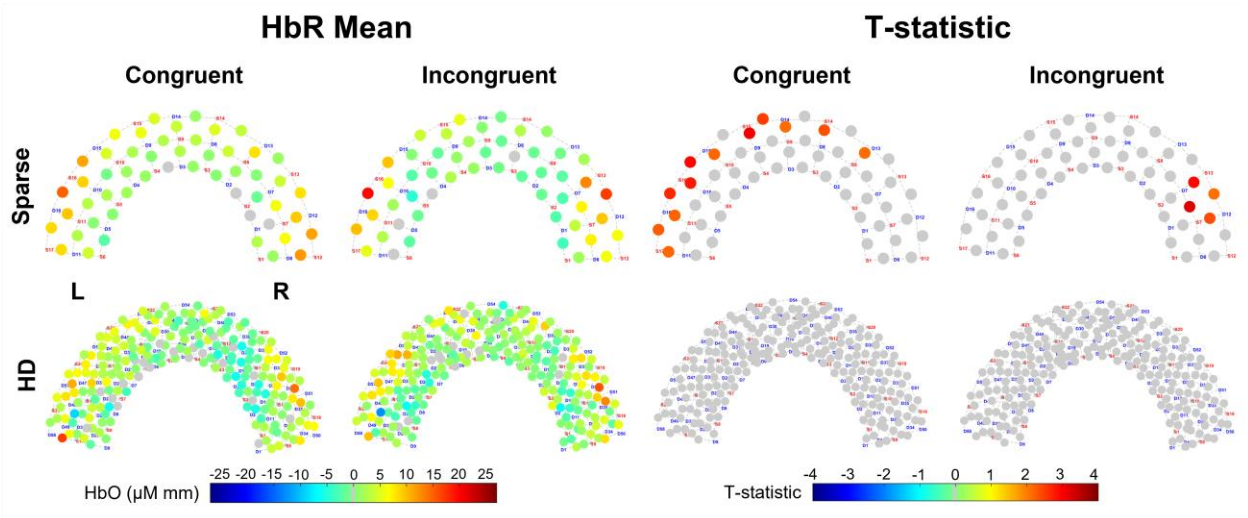

Figure S1: Channel-level brain response recorded by Sparse and HD arrays during WCS. “HbR Mean”: group-average hemodynamic response (HbR) for each channel, averaged across 7 to 18 s of the blocks for each condition. “T-statistic”: *t*-statistic of each channel across subjects is plotted. Gray channels have a p-value greater than critical p-value calculated from a cluster permutation, varies per plot.

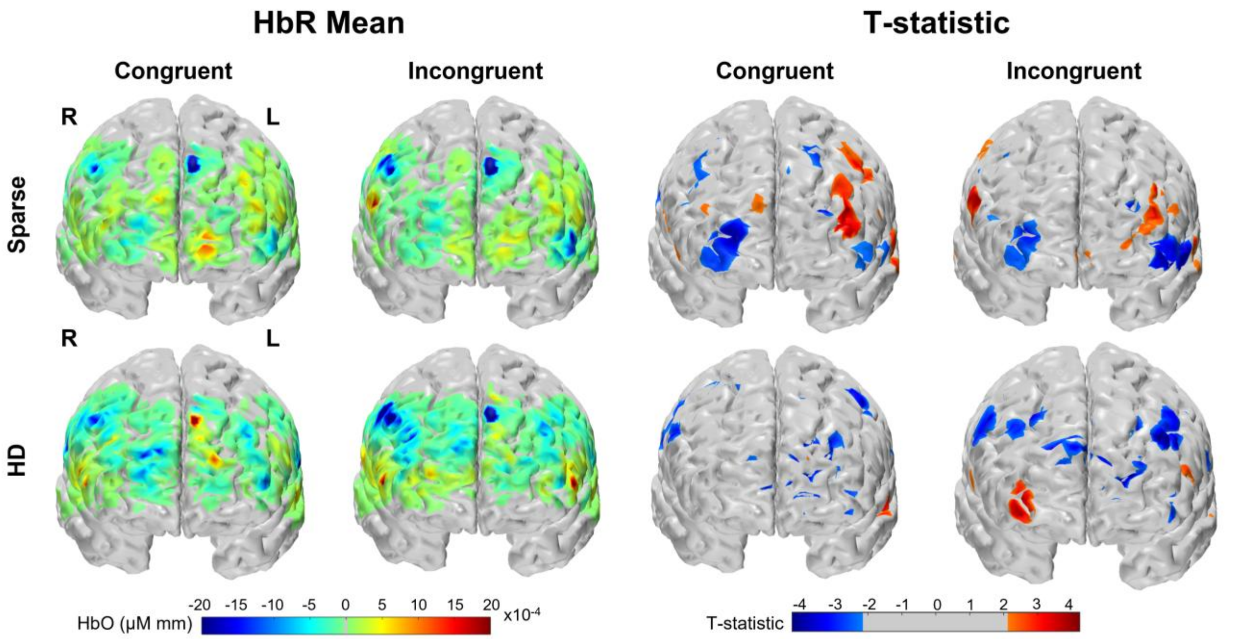

Figure S2: Brain and scalp image space brain response recorded by Sparse and HD arrays during WCS, from Anterior view. "HbR Mean": group-average hemodynamic response (HbR) for each condition. "T-statistics": *t*-statistic of each vertex across subjects is plotted. Color-scale is gray for absolute values less than two-tailed t-critical = 2.12 as calculated for 17 subjects.

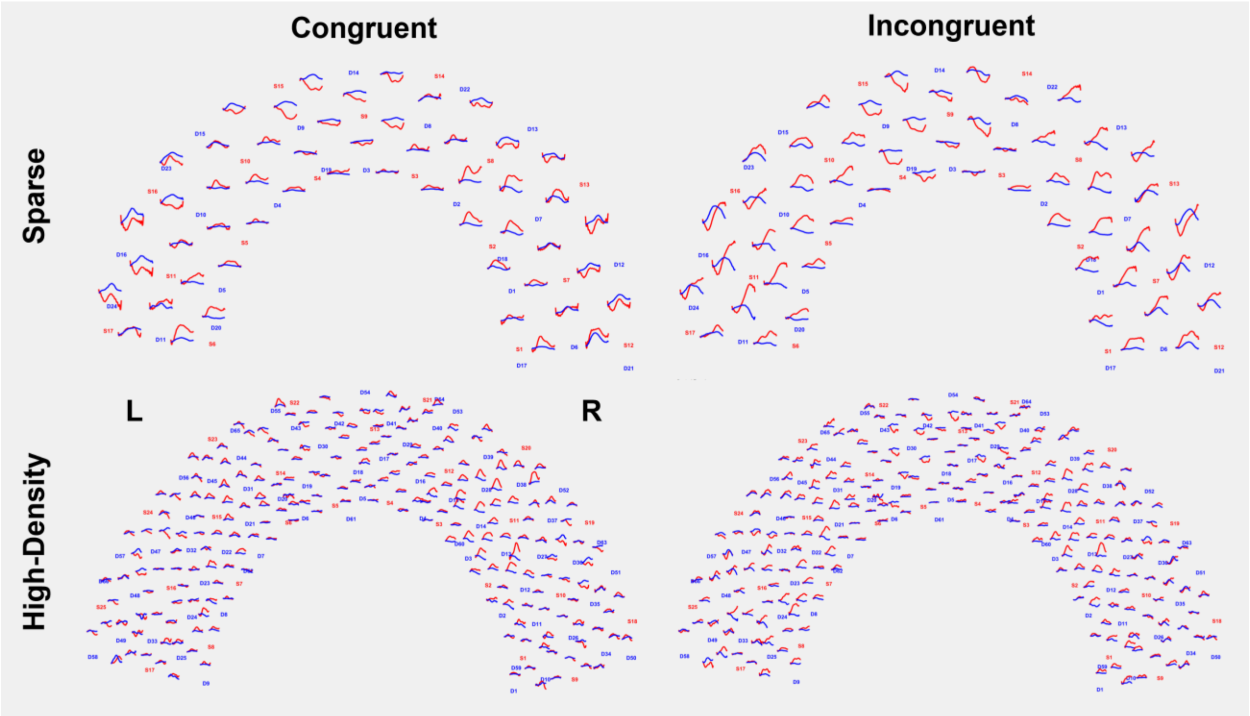

Figure S3: Group-average hemodynamic response (HbO and HbR) from -2 to 25 s for each channel. Red lines indicate HbO, blue lines indicate HbR. Y-axes agree within a result (i.e. Sparse Congruent) but are not aligned across results.

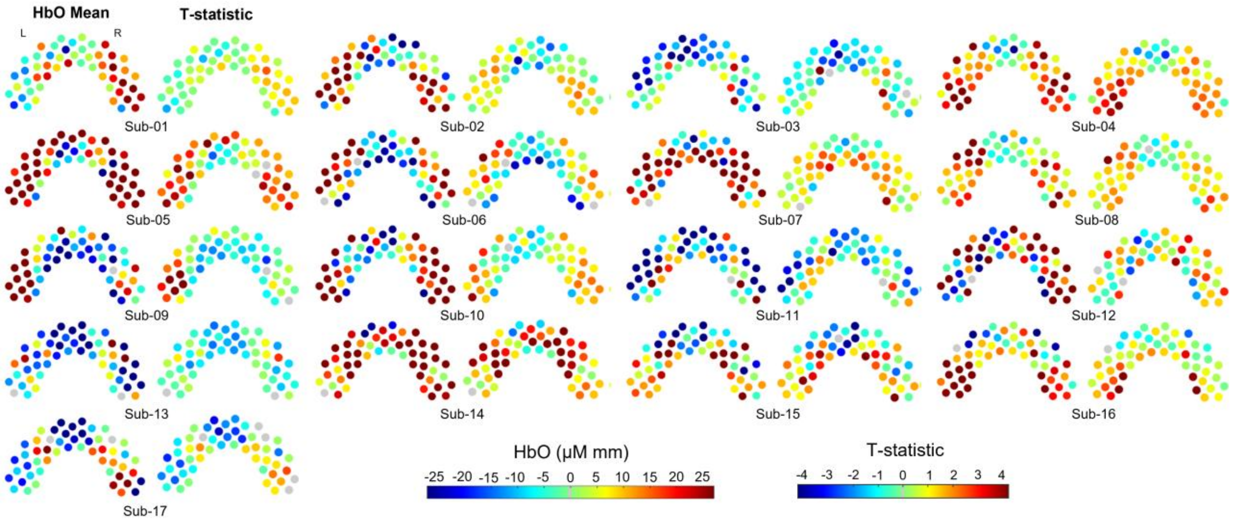

Figure S4: Channel space brain response recorded by Sparse array during Incongruent WCS, from superior view. Each subject's HbO mean and *t*-statistic across blocks is side-by-side.

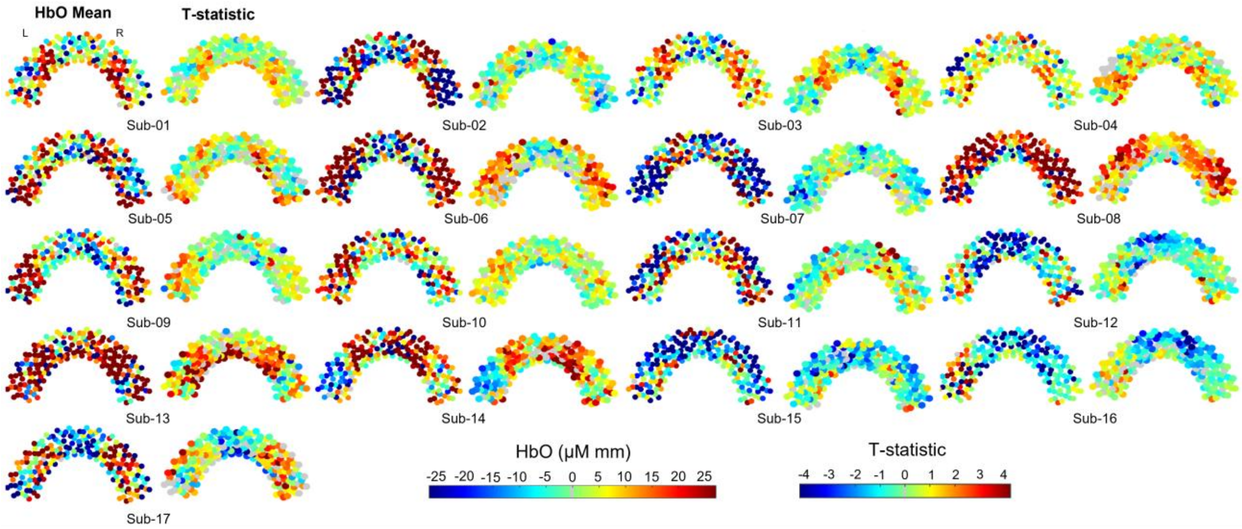

Figure S5: Channel space brain response recorded by HD array during Incongruent WCS, from superior view. Each subject's HbO mean and *t*-statistic across blocks is side-by-side.

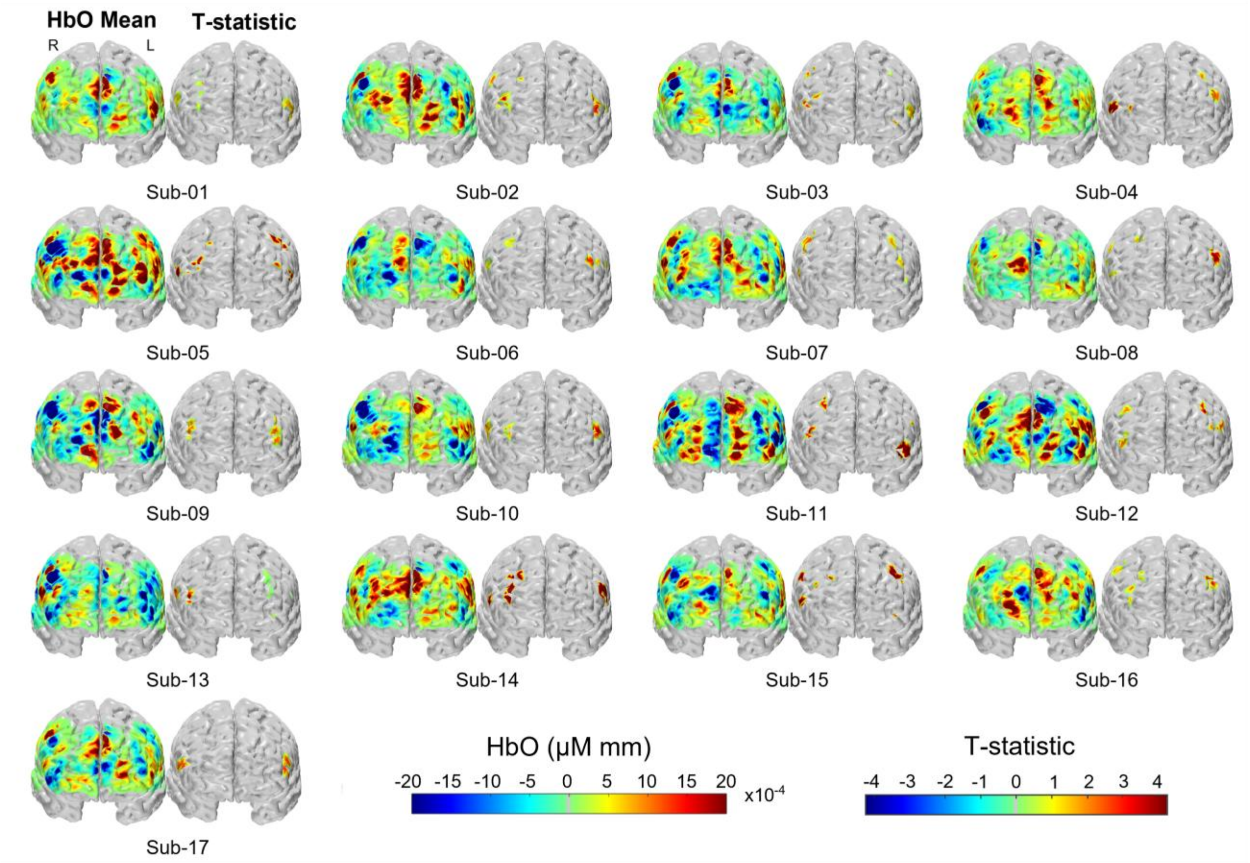

Figure S6: Brain and scalp image space brain response recorded by Sparse array during Incongruent WCS, from anterior view. Each subject's HbO mean and *t*-statistic across blocks is side-by-side. The *t*-statistic images display the top 25 vertices per ROI and were used in analysis.

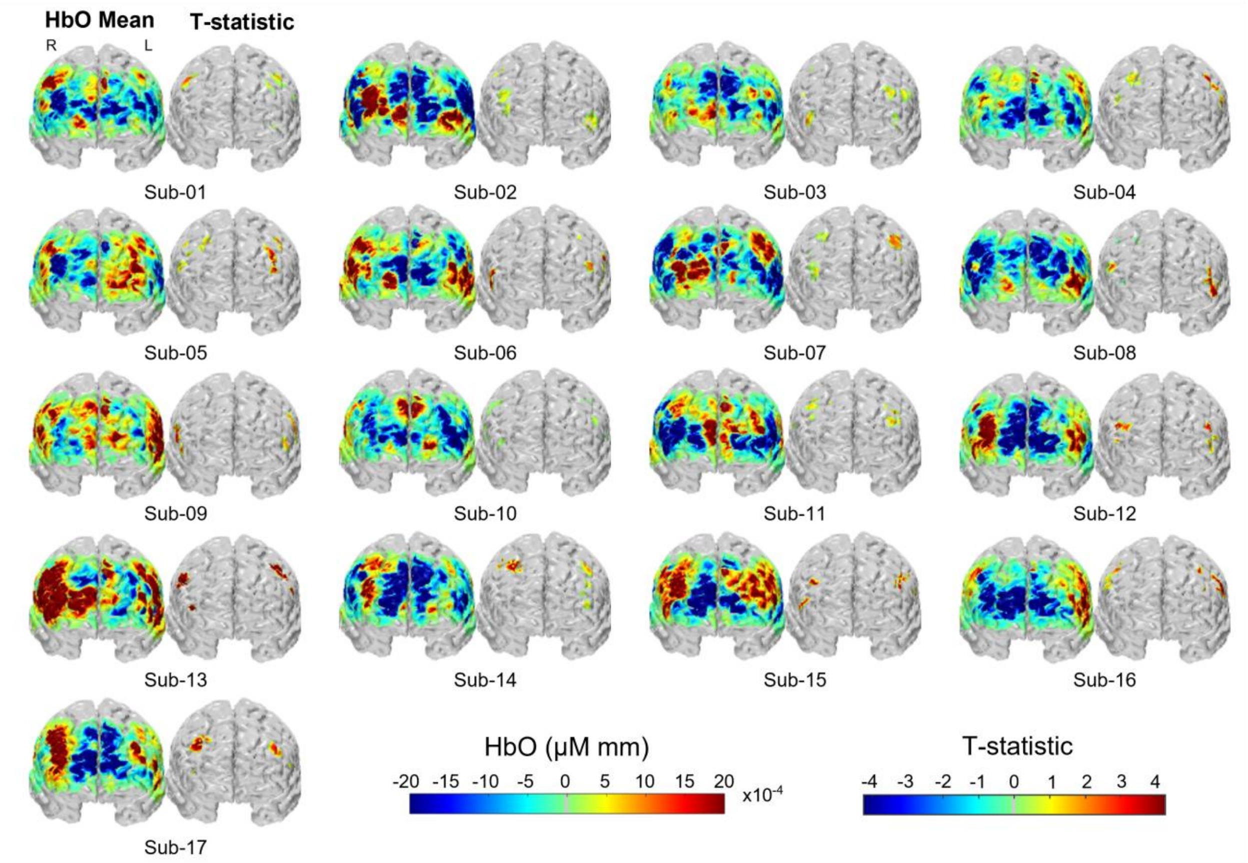

Figure S7: Brain and scalp image space brain response recorded by HD array during Incongruent WCS, from anterior view. Each subject's HbO mean and *t*-statistic across blocks is side-by-side. The *t*-statistic images display the top 25 vertices per ROI and were used in analysis.

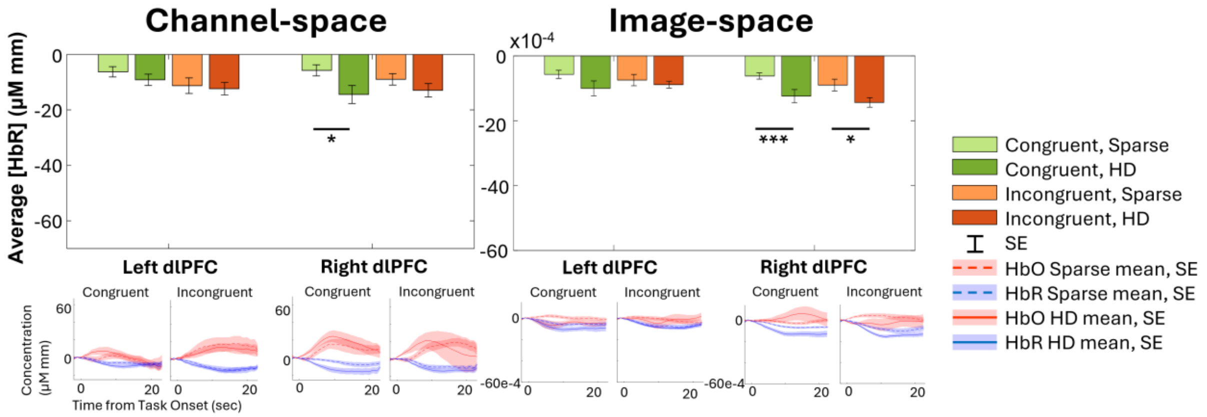

Figure S8: From within the ROIs, group-average HbR from channels or vertex clusters with minimum *t*-statistics are presented in both channel and brain and scalp image space for each array and WCS conditions. Asterisks indicate * for p ≤ 0.05, *** for p ≤ 0.001 for paired Student's *t*-test between arrays (black) and conditions (blue). The subjects' selected channel or averaged 25 vertices' concentration time courses are averaged for the timeseries plots. Numerical average, standard error, and paired Student's *t*-test values are available in Supplementary Table S1.

HbO	Channel-Space (µM mm)						Brain and Scalp Image-Space (µM mm e-4)					
	Congruent		Incongruent		**p value, Condition**		Congruent		Incongruent		**p value, Condition**	
	Left	Right	Left	Right	Left	Right	Left	Right	Left	Right	Left	Right
**Sparse**	23.71 ± 6.44	30.37 ± 6.94	38.51 ± 5.08	38.39 ± 5.62	0.118	0.371	5.88 ± 0.83	10.02 ± 1.89	7.39 ± 1.29	12.93 ± 2.21	0.220	0.170
**HD**	26.26 ± 5.78	51.20 ± 8.95	39.22 ± 5.53	40.98 ± 7.38	0.125	0.182	11.83 ± 1.98	35.64 ± 6.60	16.36 ± 2.56	39.11 ± 10.1	***≤0.001	0.603
**p_value, array**	0.771	*0.026	0.932	0.660			*0.017	***≤0.001	*0.015	*0.019		
**HbR**	**Channel-Space (-µM mm)**						**Brain and Scalp Image-Space (-µM mm e-4)**					
	Congruent		Incongruent		**p value, Condition**		Congruent		Incongruent		**p value, Condition**	
	Left	Right	Left	Right	Left	Right	Left	Right	Left	Right	Left	Right
**Sparse**	6.26 ± 1.86	5.75 ± 2.00	11.27 ± 2.82	8.98 ± 2.05	0.138	0.201	5.69 ± 1.27	6.20 ± 0.95	7.46 ± 1.75	9.00 ± 1.82	0.371	0.088
**HD**	9.15 ± 2.04	14.44± 3.27	12.36 ± 2.25	12.93 ± 2.45	0.300	0.619	10.01 ± 2.29	12.42 ± 2.05	8.87 ± 1.06	14.35 ± 1.47	0.564	0.286
**p_value, array**	0.278	*0.023	0.717	0.183			0.110	***≤0.001	0.533	*0.021		

Table S1: Corresponds to data presented in Figure 7 and Figure S8. Group-average of each subject's selected channels' or vertices' concentration for a given concentration type, WCS condition, ROI, and array. Numbers per array are the average concentration ± standard error, units of µM mm. P-value results from paired *t*-test between the arrays' selected concentration data.

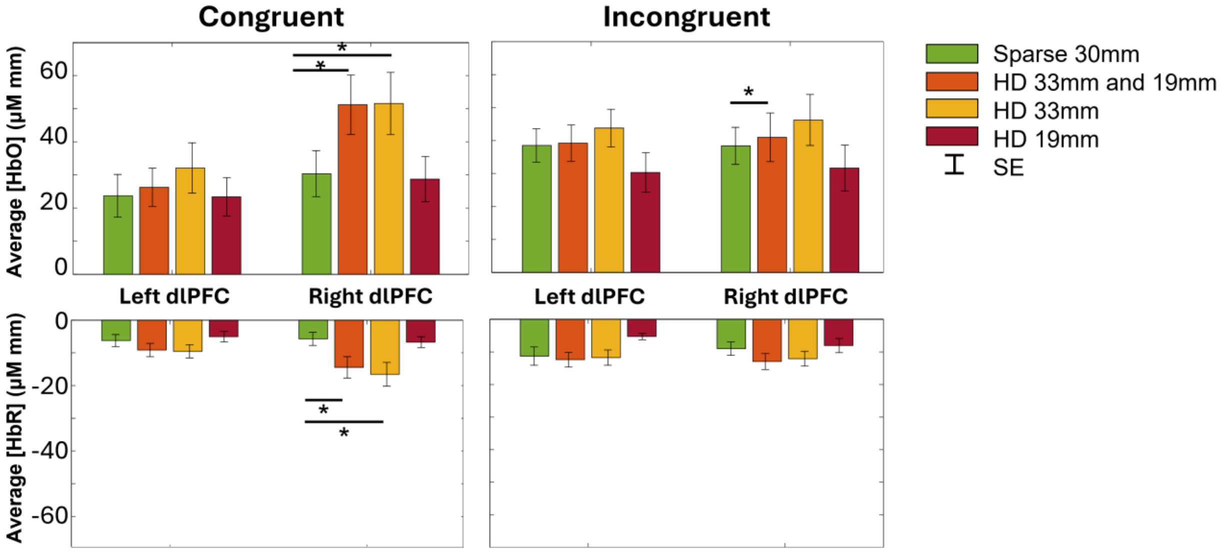

Figure S9: From within the ROIs, group-averaged maximum HbO and minimum HbR concentration from channels and vertex clusters selected for maximum and minimum *t*-statistics (respectively) are presented in channel space for each WCS condition and array channel length groups from which the channels are selected. Asterisk indicates $p \leq 0.05$ for paired Student's *t*-test between arrays. Numerical average, standard error, and paired Student's *t*-test values available in Table S2.

HbO	Channel-Space (μM mm)				HbR	Channel-Space (-μM mm)			
	Congruent		Incongruent			Congruent		Incongruent	
	Left	Right	Left	Right		Left	Right	Left	Right
**Sparse: 30mm**	23.71 ± 6.44	30.37 ± 6.94	38.51 ± 5.08	38.39 ± 5.62	**Sparse: 30mm**	6.26 ± 1.86	5.75 ± 2.00	11.27 ± 2.82	8.98 ± 2.05
**HD: 19 or 33mm**	26.26 ± 5.78	51.20 ± 8.95	39.22 ± 5.53	40.98 ± 7.38	**HD: 19 or 33mm**	9.15 ± 2.04	14.44± 3.27	12.36 ± 2.25	12.93 ± 2.45
**p_value**	0.771	*0.026	0.932	0.660	**p_value**	0.279	*0.023	0.717	0.183
**HD: 33mm**	32.11 ± 7.58	51.58 ± 9.38	43.79 ± 5.69	46.25 ± 7.74	**HD: 33mm**	9.55 ± 2.01	16.56 ± 3.63	11.74 ± 2.35	12.06 ± 2.27
**p_value**	0.422	*0.024	0.532	0.298	**p_value**	0.214	*0.020	0.876	0.232
**HD: 19mm**	23.39 ± 5.81	28.75 ± 6.84	30.32 ± 6.01	31.66 ± 6.95	**HD: 19mm**	5.07 ± 1.59	6.75 ± 1.67	5.26 ± 0.98	8.03 ± 2.19
**p_value**	0.970	0.806	0.343	0.402	**p_value**	0.628	0.578	*0.051	0.720

Table S2: Corresponds to data in Figure S9. Group average of each subject's selected channels' concentration for a given concentration type, WCS condition, ROI, and array. HD array options included when selecting from among all channels in an ROI (whether 33 mm or 19 mm), from among only the 33 mm channels in an ROI, and from among only the 19 mm channels in an ROI. Number per array are the average concentration ± standard error, units of μM mm. P-value results from paired *t*-test between arrays' selected concentration data from the Sparse and each of three HD options.

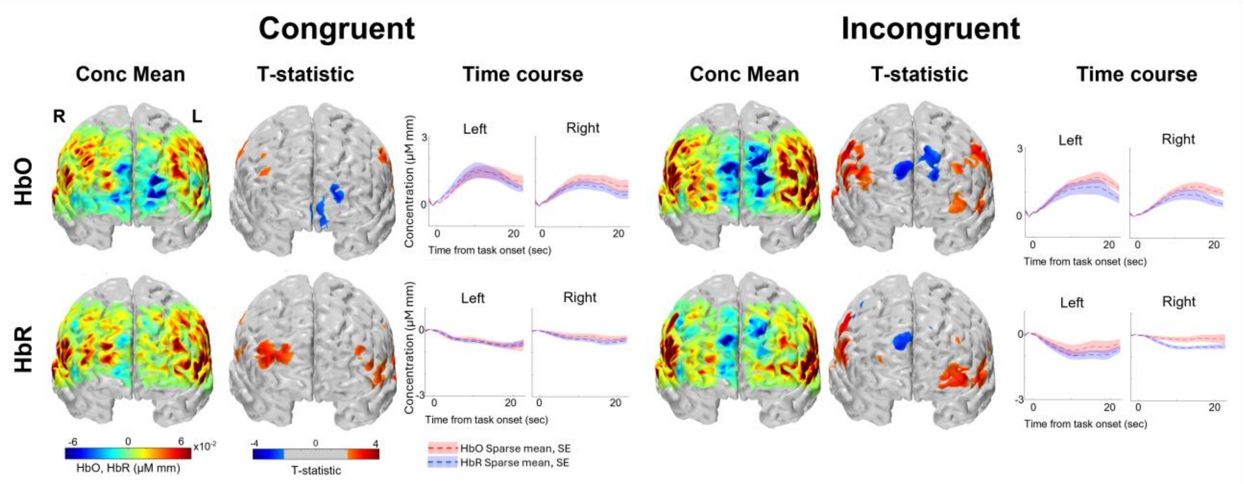

Figure S10: Performing brain only image reconstruction without regularization parameters, the image space response recorded by Sparse arrays during WCS, from anterior view. “Conc Mean”: group-average hemodynamic response for each condition. “T-statistic”: *t*-statistic of each vertex across subjects is plotted. Color-scale is gray for absolute values less than two-tailed *t*-critical = 2.12 as calculated for 17 subjects. “Time course”: the group average of each subject’s top 15 vertices by *t*-statistic ranking.

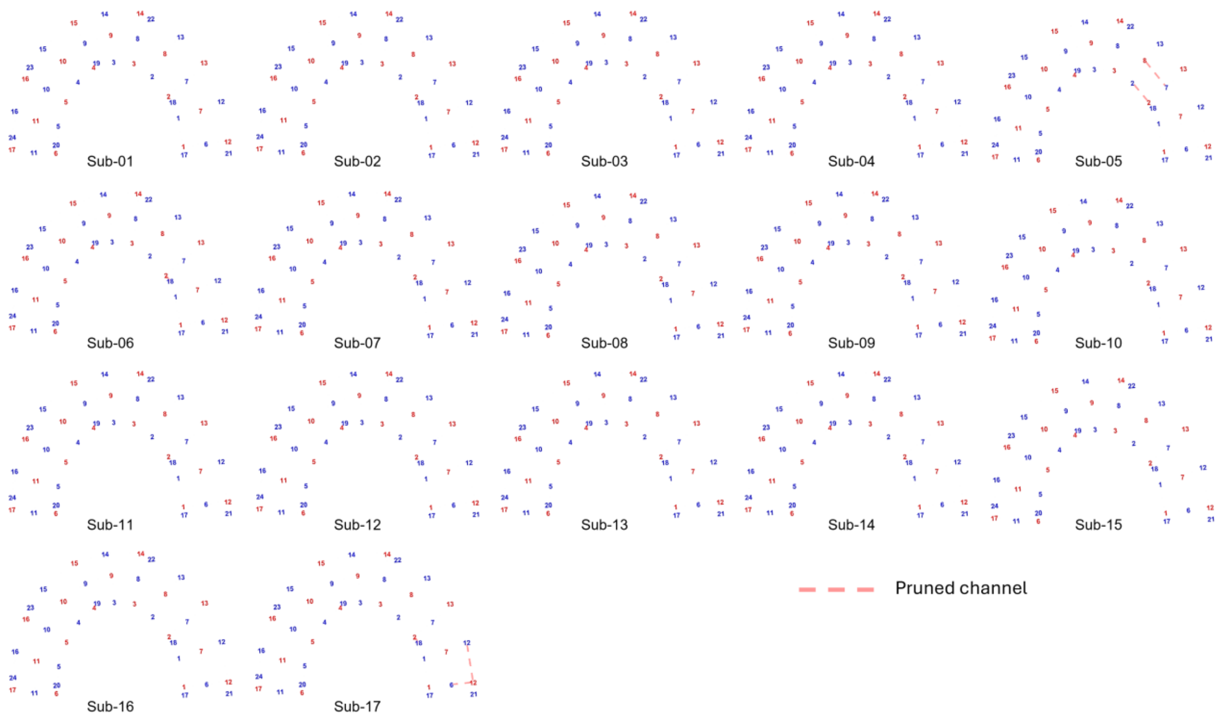

Figure S11: Visualizing subjects' pruned channels for the Sparse array, indicated by red dashed line.

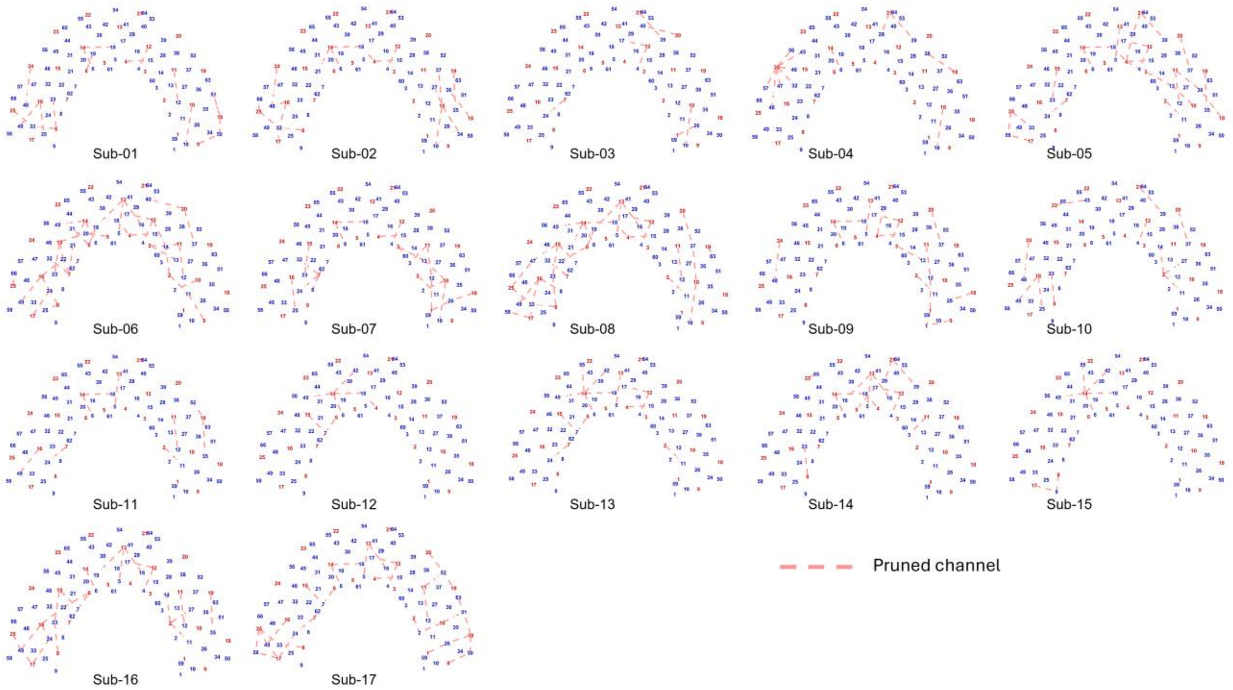

Figure S12: Visualizing subjects' pruned channels for the HD array, indicated by red dashed line.
